# Antimicrobial and immunomodulatory activity of natural bioactive compounds – an *in vitro* investigation with potential applications in managing Hidradenitis Suppurativa

**DOI:** 10.3389/fimmu.2025.1608269

**Published:** 2025-08-12

**Authors:** Valentina-Alexandra Badaluta, Adrian Ionascu, Lia-Mara Ditu, Carmen Curutiu, Alina-Maria Holban, Eliza Oprea, Yiannis Kourkoutas, Mara Madalina Mihai, Corina Ioana Cucu, Ariana Hudita, Florica Marinescu, Veronica Lazar

**Affiliations:** ^1^ University of Bucharest, Faculty of Biology, Bucharest, Romania; ^2^ Research Institute of the University of Bucharest, University of Bucharest, Bucharest, Romania; ^3^ Drosophila Laboratory, Department of Genetics, Faculty of Biology, University of Bucharest, Bucharest, Romania; ^4^ MICROGEN Research Center, Faculty of Biology, University of Bucharest, Bucharest, Romania; ^5^ Laboratory of Applied Microbiology & Biotechnology, Department of Molecular Biology & Genetics, Democritus University of Thrace, Alexandroupolis, Greece; ^6^ Department of Oncologic Dermatology, “Elias” Emergency University Hospital, Bucharest, Romania; ^7^ Department of Nephrology, Urology, Immunology and Transplant Immunology, Dermatology, and Allergology, “Carol Davila” University of Medicine and Pharmacy, Bucharest, Romania

**Keywords:** natural bioactive compounds, Hidradenitis Suppurativa, novel therapeutic strategies, anti-biofilm effects, immunomodulatory activity, probiotics

## Abstract

**Background:**

Natural bioactive compounds such as terpenoids and phenolic acids have emerged as promising agents in dermatological research due to their proven antioxidant, antimicrobial and anti-inflammatory properties. Hidradenitis Suppurativa (HS), a chronic inflammatory condition, presents a therapeutic challenge that could benefit from innovative approaches harnessing these natural compounds.

**Objective:**

The present study aimed to evaluate the antimicrobial and immunomodulatory effects of phytoconstituent agents (FCs) including Gallic Acid (GA), α–Terpineol (αT) and Nerolidol (N), both individually and in combinations. The potential of these compounds to enhance immune regulation and inhibit biofilm development in HS-related pathogen was explored through *in vitro* investigations, emphasizing their therapeutic potential in managing HS-related infections and inflammation.

**Methods:**

Phytocompounds (FCs) (GA, αT and N) were obtained by solubilization in dimethyl sulfoxide (DMSO) at an initial concentration of 10 mg/mL and tested against standard and clinical strains of HS-associated pathogens. Additionally, Lactic Acid Bacterial (LAB) strains isolated from normal microbiota, dental plaque and lactic fermented foods were assessed for their antimicrobial, anti-biofilm and immunomodulatory effect, using both qualitative and quantitative assays. The immunomodulatory properties were analyzed using macrophages differentiated from THP-1 human monocytic cells. Cytokine modulation was measured via Enzyme-Linked Immunosorbent Assay (ELISA).

**Results:**

The combination of α-terpineol and nerolidol demonstrated potent antimicrobial activity and markedly inhibiting biofilm development, particularly against Gram-positive bacterial strains. A significant modulation of the inflammatory response, including enhanced IL-10 induction, was observed when *Lactobacillus paracasei was* combined with either nerolidol or α-terpineol.

**Conclusions:**

These findings underscore the potential of natural bioactive compounds and their combinations as promising candidates for further investigation in managing skin infections and inflammation-related disorders, including HS. Future studies are essential to optimize formulations, evaluate compound stability, cytotoxicity and skin penetration and establish efficacy *in vivo*, paving the way for the development of well-tolerated and effective topical formulations.

## 1 Introduction

Resistant, multi-resistant and biofilm-forming microorganisms often cause skin infections, whose treatment represents a major challenge since they exhibit chronic evolution. Hidradenitis Suppurativa (HS) widely known as *acnea inversa* or Verneuil’s disease, is a chronic skin condition characterized by inflammatory processes, affecting various areas of the body, often rich in apocrine glands such as inguinal, lower-back, axillary, inframammary, and perianal regions. HS has been identified as a condition associated with a wide range of comorbidities (cardiovascular disease, inflammatory bowel disease, inflammatory arthritis, metabolic syndrome, non-alcoholic fatty liver disease) which led to inflammation at the level of internal organs (1–4).

The pathogenesis of HS is multifactorial and involves complex mechanisms of innate immune system: infiltration of macrophages and neutrophils, production of pro-inflammatory interleukins (IL-1, TNF-α, IL-17, IFN-γ) and B-cell mediated processes associated with antibodies production and dermal lymphatic structures (2, 5, 6).

Evan that skin commensals could produce antimicrobial peptides (AMPs) and control IL-1, IL-17A, IFN- γ synthesis (7), research studies revealed that the most common skin commensal *Staphylococcus aureus*, is implicated in the pathogenesis of HS, as a consequence of dysbiosis which generate an inflammatory maintained response (8).

A very recent study isolated and identified various bacterial strains from superficial and chronic deep HS lesions, predominantly anaerobic and facultative anaerobic strains. The heterogenicity and abundance of isolated microorganism varied depending on the severity stage of the disease, gender, body mass index and on the affected regions. *S. aureus*, *Staphylococcus epidermidis, Staphylococcus lugdunensis, Enterococcus faecalis* were the most frequently strains isolated from superficial HS lesions, meanwhile *S. aureus, S, epidermidis* and *Corynebacterium tberculostearcium* were predominant in deep lesions (8).

Moreover, an exacerbated inflammation response in damaged skin tissue, accompanied by persistent dysbiosis generates a favorable environment for superinfections which maintain chronic inflammation and delay wound healing (7).

Several potentially therapeutic strategies based on phytochemicals agents and their mechanisms of action are currently under intensive research. These include targeting the immune mediators responsible for the inhibition of pro-inflammatory cytokine synthesis and interfering with interleukin – induced signal transduction pathways and treatments targeting microbial biofilm disruption. In this context, phytocompounds have been explored as potential complementary therapeutic options for chronic skin wounds, particularly in the management of HS (9).

Natural bioactive compounds such as terpenoids and phenolic acids serve as promising therapeutic strategies for wound healing due to their proven antimicrobial, anti-inflammatory and antioxidant properties.

Gallic acid (GA) is a natural phenolic acid found in the roots, leaves and fruits of plants. Due to its proven bioavailability and bioactivity, GA has attracted researcher’s attention as a biological compound with antimicrobial and antioxidant properties. Several studies demonstrated that GA and natural derived polyGA reduced pro-inflammatory cytokines production such as: IL-6, TNF-α and IL-1β in human monocytes (10) and human keratinocytes cells (HaCaT) (11) as well as IL-17 and IFN- γ in peripheral blood mononuclear cells (PBMCs) (12). GA has been demonstrated to modulate bacterial growth, biofilm formation and virulence factors such as adherence on substrates and motility, exerting antimicrobial activity against clinically relevant MDR strains, including *Escherichia coli, Pseudomonas aeruginosa, Shigella flexneri* (13) and *Staphylococcus aureus* (14–16).

Nerolidol (N), also known as peruviol, is a sesquiterpene alcohol found in plants such as *Malaleuca* sp., *Myrocarpus fastigiatus* (17), *Myroxylon balsamum* (18), *Momordica charantia, Ginkgo biloba* (19). N possesses numerous bioactive properties including anxiolytic, larvicidal (20), antioxidant, analgesic, anti-inflammatory and antimicrobial effects (21). N’s antimicrobial activity is associated with its hydrophobic form, interacting with microbial cell membranes, compromising membrane integrity, increasing permeability, and lastly, leading to cell lysis (17, 22). Several studies have shown that this natural bioactive compound exhibited strong antimicrobial and antibiofilm effects against both Gram-negative and Gram-positive strains, including Multi-Drug Resistant (MDR) ones (19). It has been documented that N possess immunomodulatory activity by activation of human neutrophils and inhibiting FRP1/FRP2 agonist induced by neutrophil activation and chemotaxis (23). Additionally, N also demonstrates complementary effects with other bioactive compounds, enhancing its bioactivity (18, 23).

α-Terpineol (αT) is a naturally monocyclic terpene alcohol which can be found in several botanical sources, including flowers, leaves, herbs, fruits and essential oils (EOs). It has been identified in various plants such as: *Melaleuca* sp (24, 25).,, *Alpinia galanga* (26), *Amomum subulatum* (27), *Alpinia* sp (28, 29)., *Cryptocarya alba* (26) among others. αT exhibits strong antimicrobial, analgesic, antitumor, gastroprotective, cardiovascular protective, neuroprotective and anti-inflammatory effects (30), as well as inhibitory effect on biofilm development (31–34). αT showed potent antimicrobial activity against both Gram-positive and Gram-negative bacterial strains, including *Staphylococcus aureus*, *Enterococcus faecalis*, *Escherichia coli, Bacillus cereus, Helicobacter pylori* and against yeast and fungal strains such as *Candida* sp.*, Penicillium* sp., showing greater sensitivity, highlighting its promising therapeutic potential (33–35). Additionally, studies have shown that it has the ability to interfere with Quorum Sensing (QS) mechanisms and biofilm development by competitively binding transcriptional regulators, acting as a QS antagonist in *P. aeruginosa* (36).

As a therapeutic alternative, probiotics contribute significantly to the skin healing process by inhibiting pathogenic planktonic bacteria and preventing biofilm formation. They release molecules such as antimicrobial peptides, which have the ability to disrupt bacterial QS mechanisms and to prevent adhesion to the cellular substrate making them promising candidates for topical probiotic applications or oral formulations (37, 38).

Therefore, in the context of HS, systemic medical therapies or topical formulations options which could be potentially used in the management of this chronic skin condition should be effective, without negatively affecting normal gut or skin microbiota.The combination of LAB strains with polyphenols and terpenoids may provide a synergistic approach by reducing oxidative stress, modulating inflammatory processes and enhancing antimicrobial effects. This could shed light on their beneficial effects in wound healing and on their potential for developing therapeutic strategies with applications in promoting tissue repair and preventing infections, without disrupting normal microbiota.

In these circumstances, our study aimed to evaluate topical formulations combining selected natural bioactive compounds (gallic acid, nerolidol, and α-terpineol) and probiotics (e.g., *Lactobacillus paracasei*) as an alternative treatment of hidradenitis suppurativa (HS), with a focus on their antimicrobial, anti-biofilm, and immunomodulatory activities, including *in vitro* cytokine modulation.

## 2 Materials and methods

### 2.1 Chemicals

#### 2.1.1 Bioactive compounds

The phytoconstituents (FCs) represented by gallic acid (GA), Nerolidol (N) and α-Terpineol (αT), were purchased from Sigma Aldrich Chimie, Saint-Quentin-Fallavier, France.

#### 2.1.2 Samples preparation

Dimethyl sulfoxide (DMSO) (Sigma Aldrich Chimie, Saint-Quentin-Fallavier, France) was used as a solvent to improve the solubility of GA, N and αT. Solutions were prepared using both the individual phytochemicals and their binary combinations (αT:N, αT:GA and GA:N) in a 1:1 volumetric ratio as well as ternary combination (αT:N:GA) in a 1:1:1 volumetric ratio, all at an initial concentration of 10 mg/mL.

### 2.2 Microbial strains and growth conditions

Assessment of antimicrobial activity was performed using three reference strains by American Type Culture Collection (ATCC, Manassas, VA, USA): one Gram-negative bacterial strain of *Escherichia coli* ATCC 25922; two Gram-positive bacterial strains *Enterococcus faecalis* ATCC 29212 and *Staphylococcus aureus* ATCC 25923, as well as clinical, Multi-Drug Resistant (MDR) strains previously isolated from chronic skin conditions, following collaboration with Elias University Emergency Hospital, Bucharest, Romania: *Staphylococcus aureus* MRSA (Methicillin-resistant *Staphylococcus aureus*) 68, *Staphylococcus aureus* MRSA 86, *Staphylococcus aureus* 205, *Staphylococcus aureus* 216, *Enterococcus faecalis* 188, *Escherichia coli* 82 and *Klebsiella pneumoniae* 102. Additionally, four lactic acid bacterial (LAB) strains were also used: *Lactobacillus paracasei* SM4 (*Lb. paracasei*) isolated from sour cream, *Lactococcus lactis (Lc. lactis)* isolated from dental plaque, *Lactobacillus plantarum* 10 (*Lb. plantarum*) isolated from traditional sheep cheese and *Lactobacillus rhamnosus* MF9 (*Lb. rhamnosus*) isolated from new-born feces (**Table 1**). All LAB strains were cultivated in MRS (Man-Rogosa-Sharp) liquid broth medium with 1% Tween 80 in anaerobic conditions, at 30-37°C, for 24 h (39). All the strains were preserved on nutrient broth with 20% glycerol at −80°C until use, in the culture collection of the Microbiology Department of the Faculty of Biology, University of Bucharest, Romania.

**Table 1:** Codes and isolation sources of bacterial strains tested in the study.

Bacterial strains category	Bacterial strain	Code	Source, type
Gram-positive bacterial strains	*Staphylococcus aureus MRSA*	*S. aureus* MRSA 68	Isolated from HS
	*Staphylococcus aureus MRSA*	*S. aureus* MRSA 86	Isolated from HS
	*Staphylococcus aureus*	*S. aureus* 205	Isolated from HS
	*Staphylococcus aureus*	*S. aureus* 216	Isolated from HS
	*Staphylococcus aureus*	*S. aureus* ATCC 25923	Standard strain (ATCC – American Type Culture Collection)
	*Enterococcus faecalis*	*E. faecalis* ATCC 29212	Standard strain (ATCC – American Type Culture Collection)
	*Enterococcus faecalis*	*E. faecalis* 188	Isolated from HS
Gram-negative bacterial strains	*Escherichia coli*	*E. coli* ATCC 25922	Standard strain (ATCC – American Type Culture Collection)
	*Escherichia coli*	*E. coli* 82	Isolated from HS
	*Klebsiella pneumoniae*	*K. pneumoniae* 102	Isolated from HS
Lactic acid bacterial (LAB) strains	*Lactobacillus paracasei*	*Lb. paracasei* SM4	Isolated from traditional sour cream
	*Lactobacillus plantarum*	*Lb. plantarum* 10	Isolated from traditional sheep cheese
	*Lactobacillus rhamnosus*	*Lb. rhamnosus* MF9	Isolated from new-born feces
	*Lactococcus lactis*	*Lc. lactis*	Dental plaque

### 2.3 Evaluation of antimicrobial and antibiofilm activity

#### 2.3.1 Qualitative *screening* – inhibition of microbial growth

In order to determine the antimicrobial activity of FCs, we utilized an adapted method of disk diffusion assay, following the general guidelines provided in the Clinical Laboratory Standards Institute (CLSI, 2024) (40). A 0.5 McFarland suspension, corresponding to 1.5 × 10^8^ CFU (Colony Forming Units)/mL, prepared in 0.9% NaCl sterile saline solution, was used as a standardized inoculum, which was used to inoculate Petri dishes containing Mueller-Hinton (MH) agar (for bacterial strains) and MRS agar (for LAB strains). LAB strains previously grown in MRS liquid media were centrifuged twice for 3 minutes at 5000 Revolutions per minute (RPM). The sediment was resuspended in 0.9% NaCl sterile saline solution for removing the culture medium (MRS) and to obtain the standardized inoculum with 0.5 McFarland density. Sterile 6 mm diameter absorbent paper discs were disposed on the surface of Petri dishes containing MH agar (for the bacterial strains) and MRS agar (for the LAB strains), which were seeded with microbial inoculums. A volume of 10 μL of each FC solution was placed on a separate sterile disc. The Petri dishes were then incubated to allow microbial growth for 24 h at 37°C. After incubation, the diameter of growth inhibition zone (in mm) around each disc was measured.

#### 2.3.2 Quantitative *screening* – inhibition of free-floating cell development (planktonic growth)

The Minimum Inhibitory Concentration (MIC) value of the bioactive compounds was determined using a quantitative procedure based on an adapted serial microdilution standard method which was performed in Nutrient Broth liquid medium (for bacterial strains) and MRS liquid medium (for LAB strains). Sterile broth was added to 96-well microtiter plates and binary dilutions of each bioactive compound were prepared in a volume of 150 μL (with eight different concentrations: 1, 0.5, 0.25, 0.125, 0.0625, 0.03125, 0.015625 and 0.0078125 mg/mL). After the microdilutions, 15 μL of each microbial suspension with a standard density of 0.5 McFarland was added to each well and the plates were incubated for 24 h at 37°C. After incubation, the MIC values were established by visual analysis and confirmed by spectrophotometric measurement (absorbance at 620 nm) via the Biotek Synergy-HTX ELISA multi-mode reader (VT, USA) (41, 42). Each experiment was conducted in triplicate and repeated on at least three times.

#### 2.3.3 Semi-quantitative *screening* – inhibition of monospecific biofilm development (biofilm modulation)

To investigate the effect of the natural bioactive compounds on adhesion and biofilm formation, a semi-quantitative assessment was performed using the crystal violet microdilution method. 96 well microtiter plates were prepared with binary dilutions of the FCs tested in a final volume of 150 μL of sterile broth. Each well was inoculated with 15 μL of each microbial suspension at 1.5 × 10^8^ CFU mL prepared in sterile saline. The plates were incubated for 24 h at 37°C and after incubation, the liquid medium in the wells was carefully removed, and the wells were washed three times with sterile saline to remove unattached cells. The microbial cells adhered to the well surfaces were fixed with cold methyl alcohol/methanol for 5 minutes. After removing the methanol, the plates were stained with 1% crystal violet solution for 20 minutes and the excess dye was washed off with tap water. The appearance of the biofilms developed on the inert substrate was analyzed using an inverted microscope (40X). The stained biofilms were resuspended with a 33% acetic acid solution and the absorbance of the resulting suspensions was measured spectrophotometrically at 492 nm using an ELISA multi-mode reader (41, 43, 44). Each experiment was conducted in triplicate and repeated at least three times.

### 2.4 Evaluation of the cytokines immunomodulatory pattern

#### 2.4.1 Monocyte isolation and macrophages differentiation

The THP-1 human leukemia monocytic cell line was purchased from the American Type Culture Collection (ATCC). Cells were maintained in a culture medium (Roswell Park Memorial Institute Medium – RPMI) 1640 supplemented with 10% Fetal Bovine Serum (FBS), antibiotics: 100U/mL penicillin, 100 μg/mL streptomycin and 2 mM glutamine) at 37°C in a humidified atmosphere with 5% CO_2,_ following the supplier’s recommendations. For the assessment of immunomodulatory effects, the cells were seeded into 24-well plates at a concentration of 1 x 10^6^ cells/mL in a volume of 1 mL per well. Differentiation into macrophages was induced by treating the cells with 100 ng/mL of Phorbol 12-Myristate 13-Acetate (PMA) for 72 h (45).

After incubation, the medium containing PMA was removed and cells were washed twice with PBS and the differentiated cells were maintained in RPMI 1640 supplemented with 10% FBS, without PMA. for 48 h, before experimental infection.

#### 2.4.2 Cytokines secretion induction

THP-1 cells differentiated into macrophages were infected with bacterial suspensions at a standard density of 1–3 × 10^7^ CFU/mL. The infection conditions were as follows: 450 μL RPMI medium and 50 μL microbial suspension. The bacterial strains used included Gram-positive strains: *S. aureus* MRSA 86, *S. aureus* ATCC 25923, *E. faecalis* 188 and *E. faecalis* ATCC 29212; Gram-negative strains: *E. coli* 82, *E. coli* ATCC 25922, *K. pneumoniae* 102 (representing bacterial strains control) and LAB strains: *Lb. paracasei*, *Lb. rhamnosus* and *Lc. lactis* (representing LAB control).

Decimal dilutions of the selected FCs solutions were prepared in DMSO to obtain subinhibitory concentrations (0.01 mg/mL). Cell cultures were inoculated with the final solutions, which included the following: compound control – 450 μL RPMI medium and 50 μL of the natural bioactive compound (Gallic Acid (GA), Nerolidol (N) or α-Terpineol (αT)) at a concentration of 0.01 mg/mL; the working suspensions (infected with pathogens or exposed to LAB strains and treated with FCs at subinhibitory concentrations) – 400 μL RPMI medium, 50 μL of the natural bioactive compound (G, N or αT) at a 0.01 mg/mL concentration, and 50 μL of the microbial suspension containing the following strains: Gram-positive strains (*S. aureus* MRSA 86, *S. aureus* ATCC 25923, *E. faecalis* 188 and *E. faecalis* ATCC 29212), Gram-negative strains (*E. coli* 82, *E. coli* ATCC 25922, *K. pneumoniae* 102) and LAB strains (*Lb. paracasei*, *Lb. rhamnosus* and *Lc. lactis*).

We selected a sub-inhibitory concentration of 0.01 mg/mL for the phytoconstituents, to ensure that the observed effects on cytokine production were not confounded with antimicrobial activity. This dose is below the MIC values determined for the tested isolates and is consistent with concentrations reported in the literature (20 – 100 µg/mL) for assessing immune modulation without inducing cytotoxicity (19, 46, 47).

The plates were incubated for 3 h at 37°C in a humidified atmosphere, with 5% CO_2_. After incubation, cell supernatants were collected into Eppendorf tubes and stored at −20°C. The cell cultures were then washed twice with PBS to remove non-internalized microorganisms and reinoculated with 500 μL of RPMI medium supplemented with FBS to continue incubation for 24 h and 48 h, ensuring that cell viability was not affected by dehydration.

Throughout the incubation period, cell cultures were analyzed in real time using an inverted microscope.

After the 24 h and 48 h stimulation periods, cell supernatants were collected into Eppendorf tubes and stored at −20°C. The cell cultures were gently washed twice with PBS and fixed with 500 μL of cold methanol for 5 minutes. After removing the methanol, the plates were stained with May-Grünwald Giemsa solution for 20 minutes. Following stain removal, the plates were air-dried at room temperature, and the morphology of macrophages post-infection was analyzed using an inverted microscope.

#### 2.4.3 Enzyme-linked immunosorbent assay

The production of pro-inflammatory (TNF-α, IL-2, IL-6, IL-12A) and anti-inflammatory (IL-10) interleukins was measured using commercial ELISA kits purchased from EIAab Science INC, Wuhan, China, according to the manufacturer’s instructions.

The experiments were performed on cell supernatants obtained after the experimental infection of differentiated macrophages and treatment with subinhibitory concentrations of FCs: GA, N and αT (0.01 mg/mL), following 24 h and 48 h of stimulation.

Before analysis by ELISA, the cell supernatants were centrifuged at 10–000 RPM for 5 minutes. According to ELISA kit protocols, the results were interpreted after spectrophotometer readings at 450 nm, using the Biotek Synergy-HTX ELISA multi-mode reader (VT, USA).

### 2.5 Statistical analysis

All measurements within section 3.1 (Evaluation of antimicrobial and antibiofilm activity of FCs) have been recorded in triplicates (n=3). Due to the uniformity of the data, with all recordings having identical values, negligible variation was consistently added to the third biological replicate only (0.001 – 0.1), in order to perform statistical analysis. Variation was not added selectively to not artificially benefit a certain category of interest but was added to the third replicate regardless if the replicate data was perfect, or variation existed in the raw data. No statistically significant differences were found between the raw data and the slightly modified data (**Supplementary Table**). The results corresponding to the evaluation of antimicrobial activity were analyzed using unpaired Stuent’s t-tests between each experimental condition or ANOVA with *post-hoc* Tukey’s multiple comparisons test.

Experiments presented in section 3.2 (Evaluation of the cytokines immunomodulatory pattern) comprised of a series of ELISA plates, one for each cytokine of interest. Simple linear regressions and their corresponding formulas were implemented for each cytokine of interest, in accordance with the protocol provided by the manufacturer. Cytokine concentrations were calculated by formula implementation and negative concentrations were removed. Mean concentrations of Gram-positive strains or Gram-negative strains were calculated for each treatment type (n = 1). The control concentration (cellular substrate alone) was subtracted from the calculated means. While assessing the cytokine concentrations in Lactobacilli strains, treatment effects were evaluated on each strain (n = 1).

Statistical significance was considered at threshold α = 0.05. All graphical representations were created in GraphPad Prism 8.4.2 software (GraphPad Prism version 8.4.2 for Windows, GraphPad Software, Boston, Massachusetts USA, www.graphpad.com).

## 3 Results

### 3.1 Evaluation of antimicrobial and antibiofilm activity of FCs

#### 3.1.1 Inhibition of microbial growth

**Figure 1:**
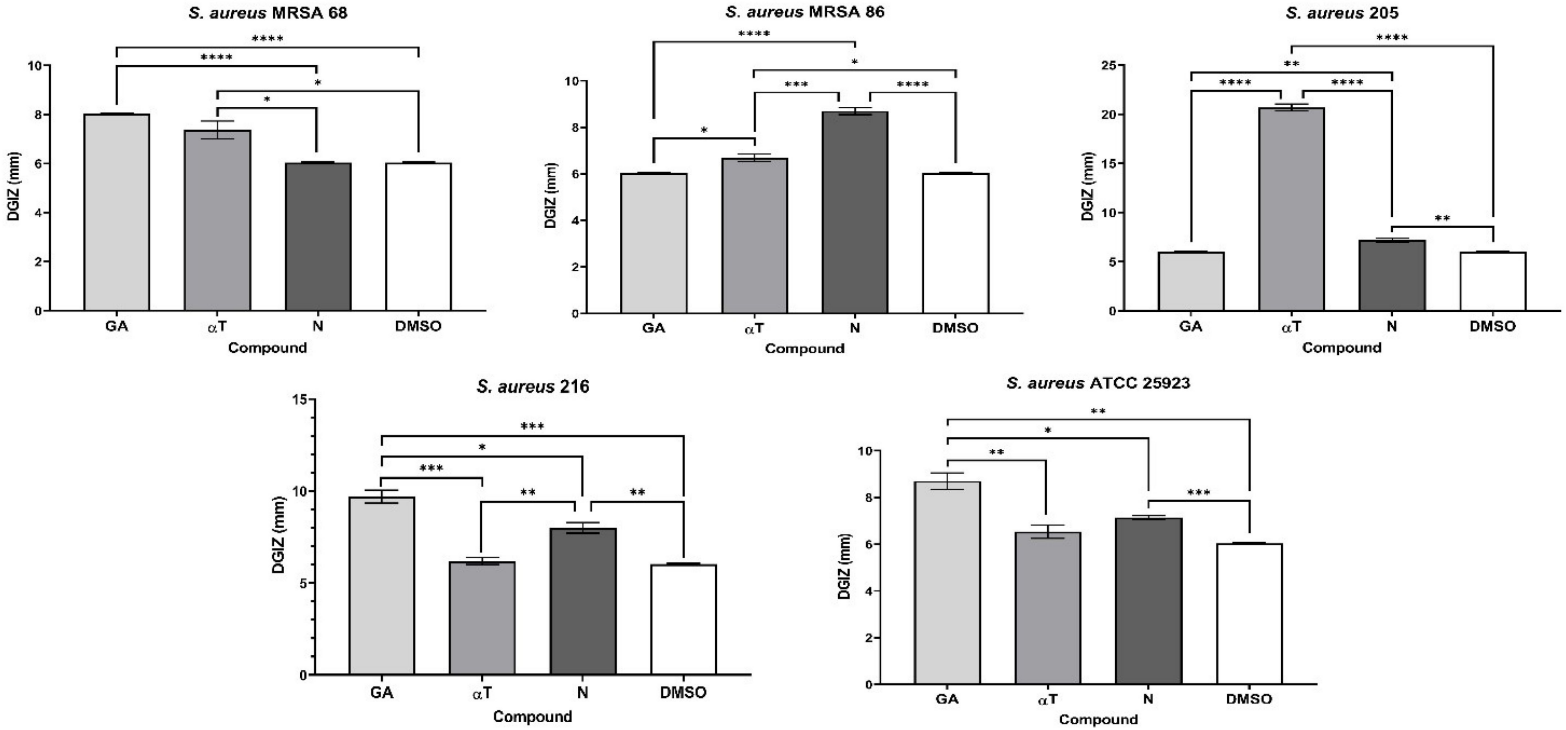
Graphic representation of Diameters of Growth Inhibition Zones (DGIZ) (mm) developed after 24 h of incubation of *S. aureus* strains (Gram-positive) in the presence of FCs: Gallic Acid (GA), α-Terpineol (αT) and Nerolidol (N) (*p < 0.05; **p < 0.01, ***p < 0.001, ****p < 0.0001). Barplots were created with modified data.

**Figure 2:**
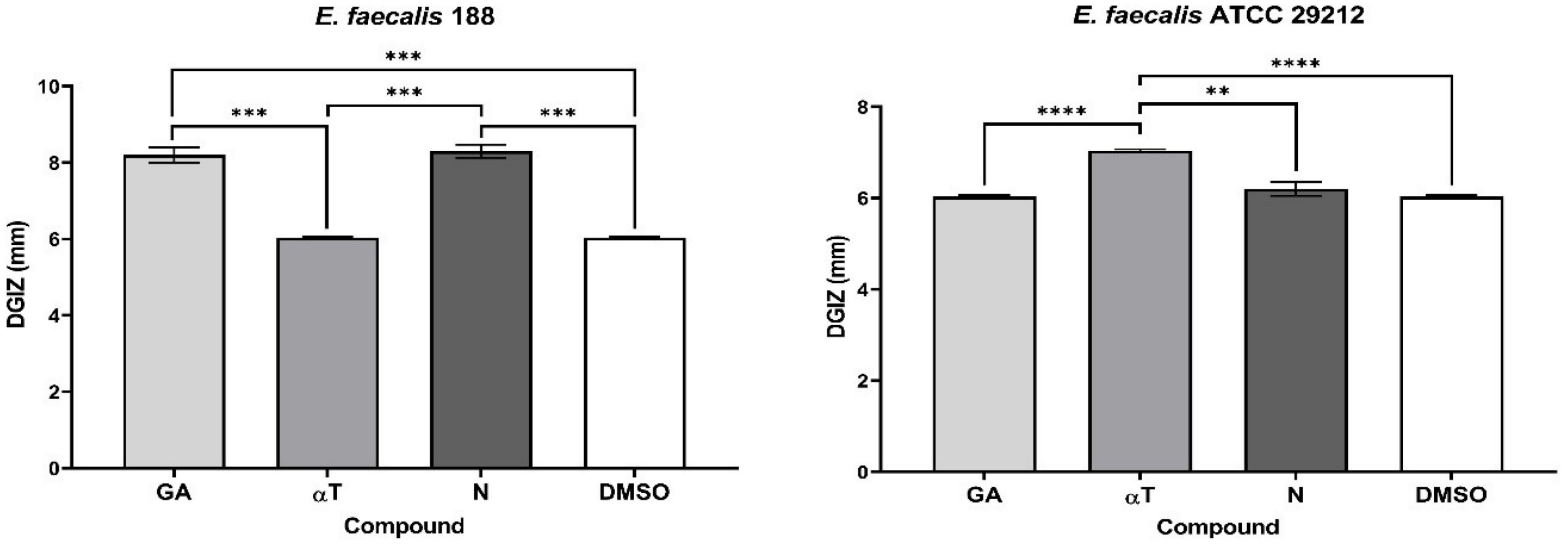
Graphic representation of Diameters of Growth Inhibition Zones (DGIZ) (mm) developed after 24 h of incubation of *E. faecalis* strains (Gram-positive) in the presence of FCs: Gallic Acid (GA), α-Terpineol (αT) and Nerolidol (N) (**p < 0.01, ***p < 0.001, ****p < 0.0001).

**Figure 3:**
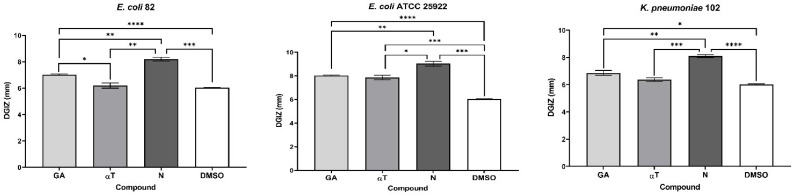
Graphic representation of Diameters of Growth Inhibition Zones (DGIZ) (mm) developed after 24 h of incubation of *E. coli* and *K. pneumoniae* strains (Gram-negative) in the presence of FCs: Gallic Acid (GA), α-Terpineol (αT) and Nerolidol (N) (*p < 0.05; **p < 0.01, ***p < 0.001, ****p < 0.0001). Barplots were created with modified data.

The qualitative analysis of the tests showed that some of bioactive FCs tested exhibited a selective inhibitory effect, in a strain-dependent manner. In Gram-positive bacterial strains, variation in antimicrobial effect among different strains was observed: a moderate inhibitory effect was generally noted, with a slight enhancement in growth, except for the *S. aureus* 205 strain which exhibited a significantly stronger inhibitory effect in the presence of αT (Diameter of Growth Inhibition Zone (DGIZ) – 20 mm) (**Figures 1**, **2**). The behavior of Gram-negative strains was more homogeneous, with most exhibiting inhibition zone with DGIZ ranging between 6 and 9 mm, generally reduced than those of Gram-positive strains (**Figure 3**). In addition, N exhibited a moderate effect bacterial growth (DGIZ ranging between 6 and 9 mm). The LAB strains exhibited the smallest DGIZ, measuring 6 mm diameters.

In the case of using binary and ternary combinations of the three natural bioactive compounds, the qualitative results did not show significantly larger growth inhibition zones compared to the previously presented qualitative tests for Gram-positive bacterial strains.

**Figure 4:**
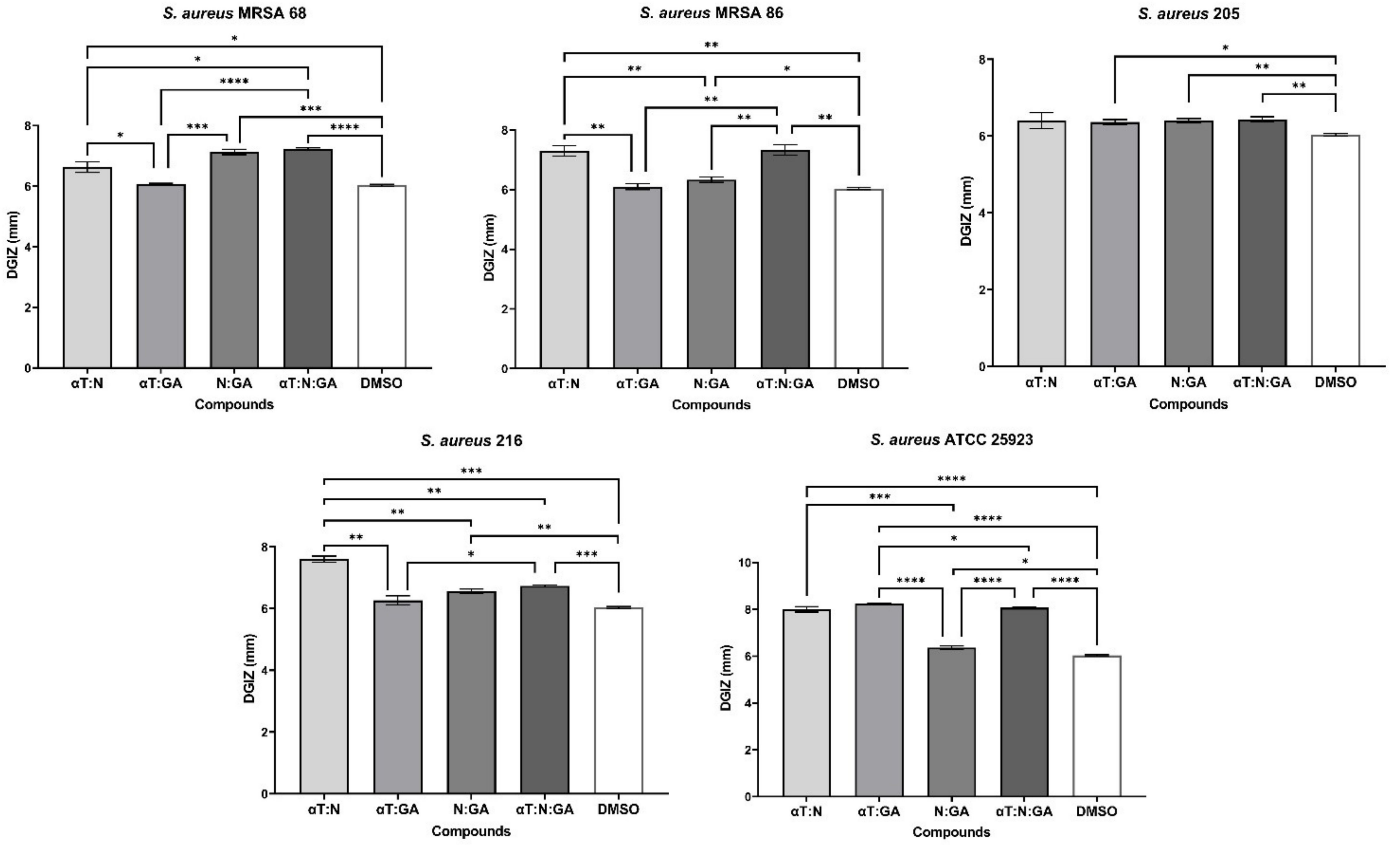
Graphic representation of Diameters of Growth Inhibition Zones (DGIZ) (mm) developed after 24 h of incubation of *S. aureus* strains (Gram-positive) in the presence of binary combinations (αT:N, αT:GA and GA:N) in a 1:1 volumetric ratio as well as ternary combination (αT:N:GA) in a 1:1:1 volumetric ratio, all at an initial concentration of 10 mg/mL. (*p < 0.05; **p < 0.01, ***p < 0.001, ****p < 0.0001). Barplots were created with modified data.

**Figure 5:**
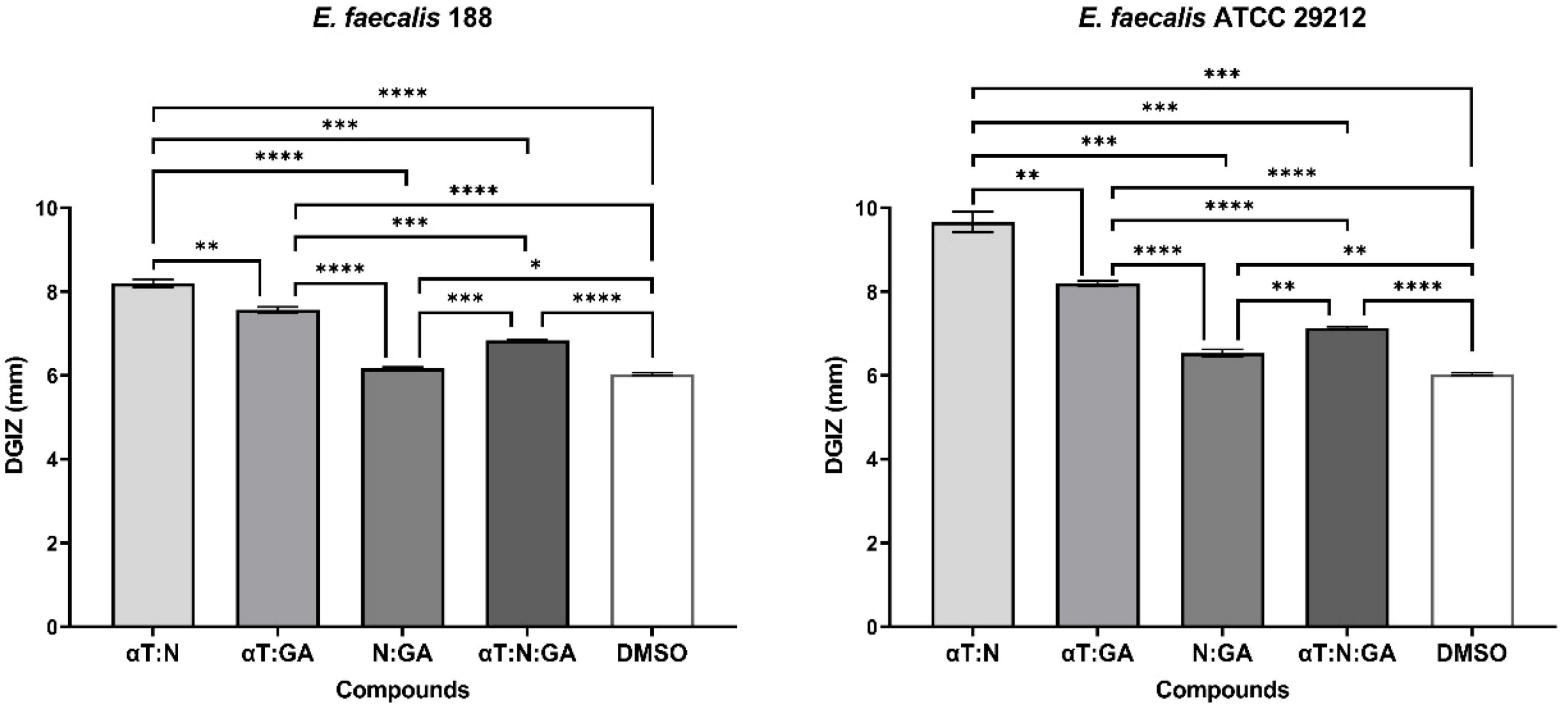
Graphic representation of Diameters of Growth Inhibition Zones (DGIZ) (mm) developed after 24 h of incubation of *E. faecalis* strains (Gram-positive) in the presence of binary combinations (αT:N, αT:GA and GA:N) in a 1:1 volumetric ratio as well as ternary combination (αT:N:GA) in a 1:1:1 volumetric ratio, all at an initial concentration of 10 mg/mL. (*p < 0.05; **p < 0.01, ***p < 0.001, ****p < 0.0001). Barplots were created with modified data.

**Figure 6:**
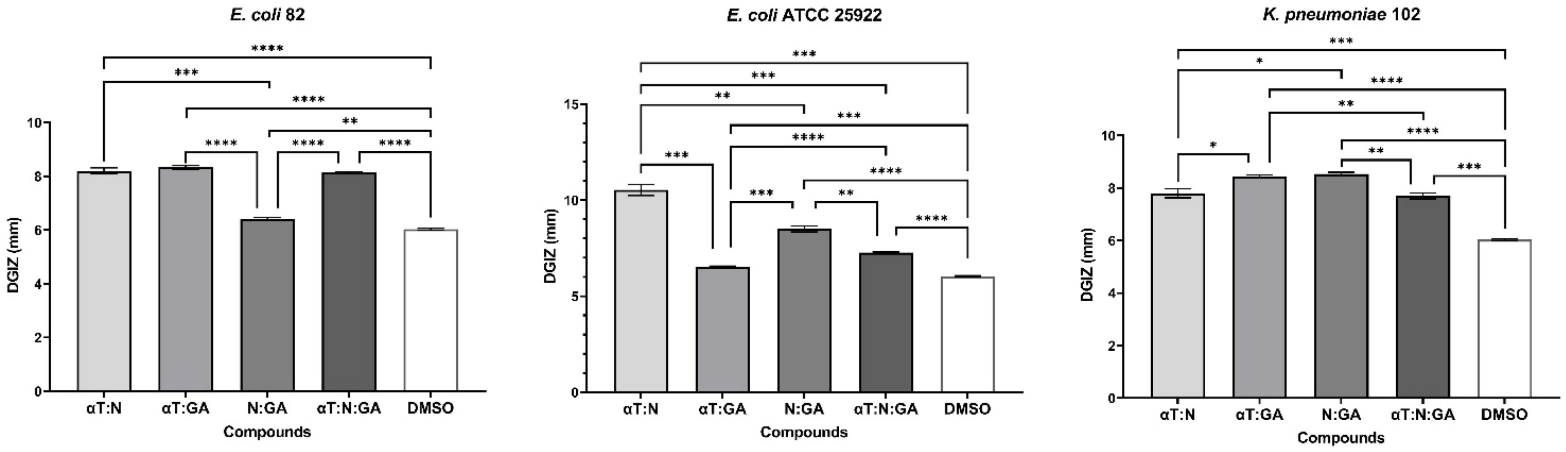
Graphic representation of Diameters of Growth Inhibition Zones (DGIZ) (mm) developed after 24 h of incubation of *E. coli* and *K. pneumoniae* strains (Gram-negative) in the presence of binary combinations (αT:N, αT:GA and GA:N) in a 1:1 volumetric ratio as well as ternary combination (αT:N:GA) in a 1:1:1 volumetric ratio, all at an initial concentration of 10 mg/mL. (*p < 0.05; **p < 0.01, ***p < 0.001, ****p < 0.0001). Barplots were created with modified data.

The significant inhibitory effect of αT on the *S. aureus* 205 strain was neutralized when combined with the other two compounds (**Figures 4**, **5**). For Gram-negative strains, DGIZ ranging between 7 and 11 mm were measured, with slightly larger diameters observed in the αT:N combination (1:1), which was selected for quantitative antimicrobial tests (**Figure 6**).

In the case of LAB strains, DGIZ was equal to that of the sterile absorbent paper discs (6 mm) in the presence of FCs.

#### 3.1.2 Inhibition of free-floating cell development (planktonic growth)

The results of the quantitative assays revealed that tested natural bioactive compounds exhibited different antimicrobial effects depending on the tested strain and concentration.

**Figure 7:**
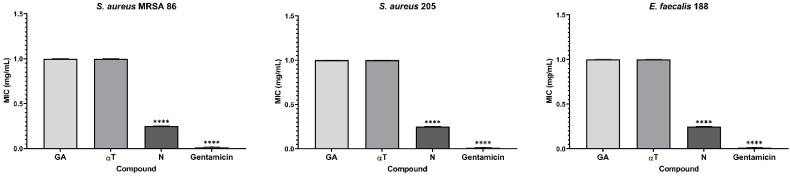
Graphic representation of the MIC values (mg/mL) after 24 h of incubation of microbial strains in the presence of FCs (****p < 0.0001). Barplots were created with modified data.

A noticeable inhibitory effect on the growth and multiplication of bacterial cells in suspension was observed, particularly in Gram-positive species. The minimum inhibitory concentrations (MIC) assay revealed that the most effective compound against Gram-positive strains was N (mean MIC – 0.25 mg/mL) for three out of the seven tested strains: *S. aureus* MRSA 86, *S. aureus* 205 and *E. faecalis* 188 (**Figure 7**).

However, although less pronounced, the inhibition of planktonic growth in Gram-negative strains remains consistent for all the FCs assessed (mean MIC – 1 mg/mL). The solvent control samples (DMSO) showed no antimicrobial activity against the tested microbial strains (mean MIC values > 1 mg/mL). For LAB strains, the MIC values exceeded 1 mg/ml, confirming the results of the qualitative tests.

Based on the results obtained from the qualitative assay, the αT:N (1:1) combination exhibited notable antimicrobial activity against both Gram-positive and Gram-negative bacterial strains. Therefore, this combination was selected for the quantitative assessment to explore its complementary effects and potential for enhanced efficacy.

**Figure 8:**
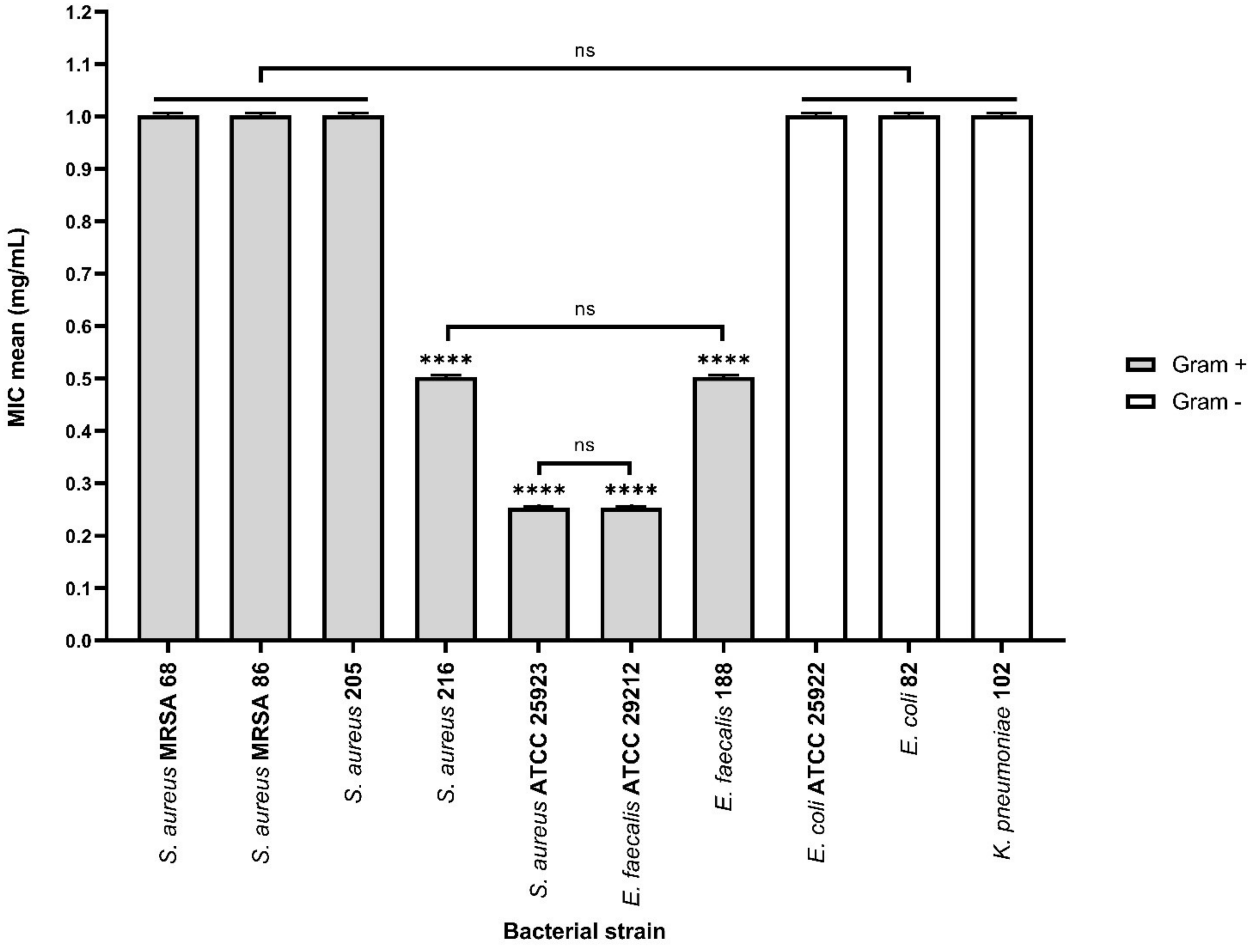
Graphic representation of the MIC values (mg/mL) after 24 h of incubation of microbial strains in the presence of αT:N (1:1) combination (ns – (not significant) *p* > 0.05; ****p < 0.0001). Barplots were created with modified data.

αT:N (1:1) combination demonstrated its effectiveness against the multiplication of Gram-positive cells in suspension. Reduced MIC values (0.25–1 mg/mL) were obtained for four out of the seven tested Gram-positive strains, such as *S. aureus* 216, *S. aureus* ATCC 25923, *E. faecalis* ATCC 29212 and *E. faecalis* 188, showing a significantly improved result compared to the individual testing of the two compounds and the qualitative tests (**Figure 8**).

On the other hand, the antimicrobial activity against Gram-negative bacterial strains was not enhanced by using the combination of the two compounds, as the MIC values were greater than or equal to 1 mg/mL.

#### 3.1.3 Inhibition of monospecific biofilm development (biofilm modulation)

The evaluation of biofilm development on an inert substrate using the crystal violet staining method enabled the determination of the Minimum Biofilm Eradication Concentration (MBEC).

**Figure 9:**
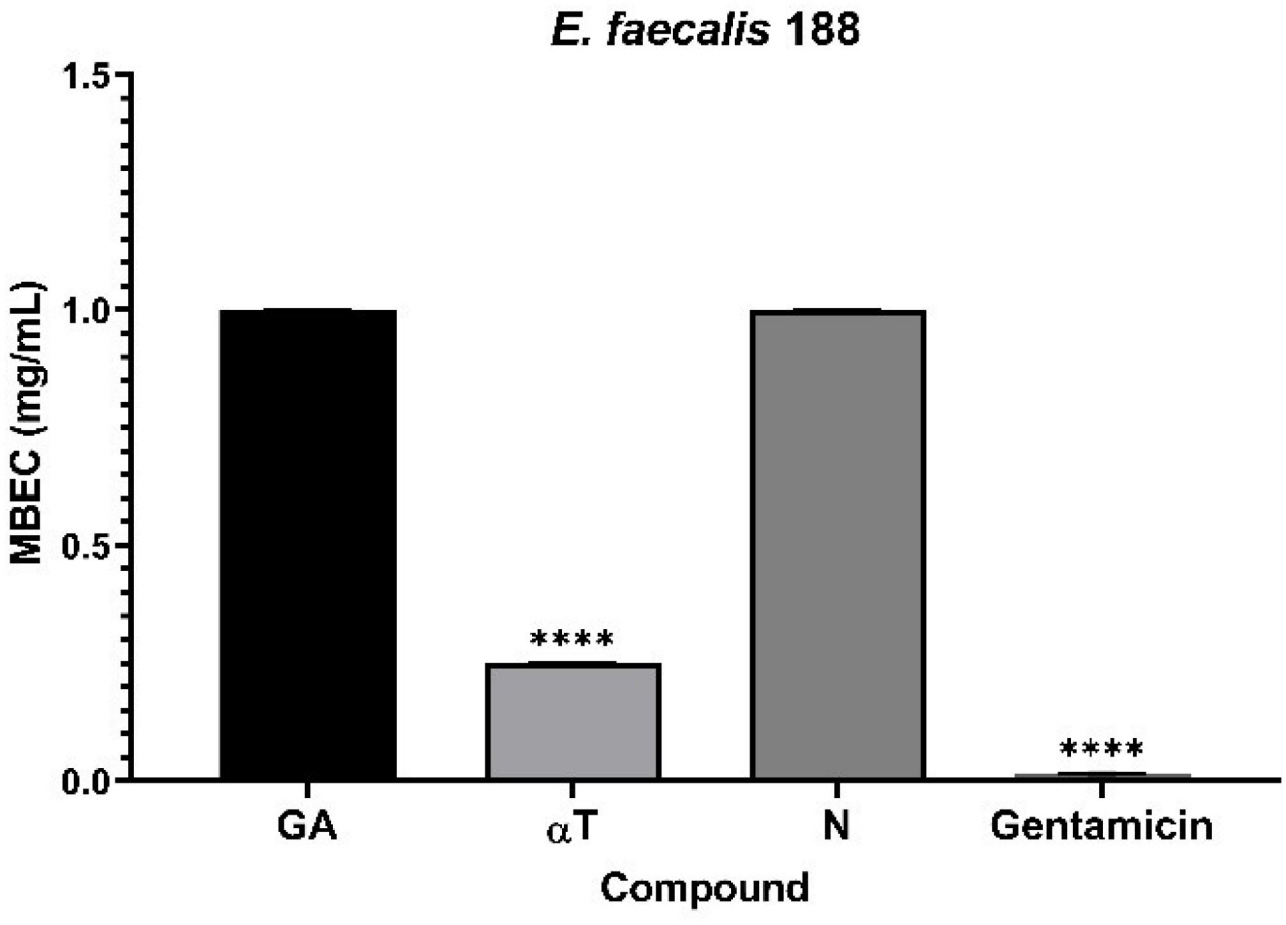
Graphic representation of biofilm modulation of antimicrobial activity of αT, N and GA for *E. faecalis* 188, after 24 h incubation; (****p < 0.0001). Barplots were created with modified data.

It was observed that monospecific biofilm formation on an inert substrate was altered in a strain-specific and bioactive compound dependent manner. Monospecific biofilm development was only slightly inhibited in the presence of N, specifically in the case of the clinical *E. faecalis* strain (mean MBEC – 0.25 mg/mL) (**Figure 9**). In contrast, the tested compounds exhibited low anti-biofilm activity against the other microbial strains (mean MBEC – 1 mg/mL).

On the other hand, the effect of bioactive compounds on biofilm formation was more efficient in the presence of the αT:N combination in a 1:1 volumetric ratio, for most implicated wound biofilm opportunistic pathogens, both standard and clinical resistant strains isolated from HS.

For Gram-positive bacteria, the results indicated that the biofilm development was altered in the presence of the selected combination, with mean MBEC values between 0.0625 and 0.125 mg/mL.

**Figure 10:**
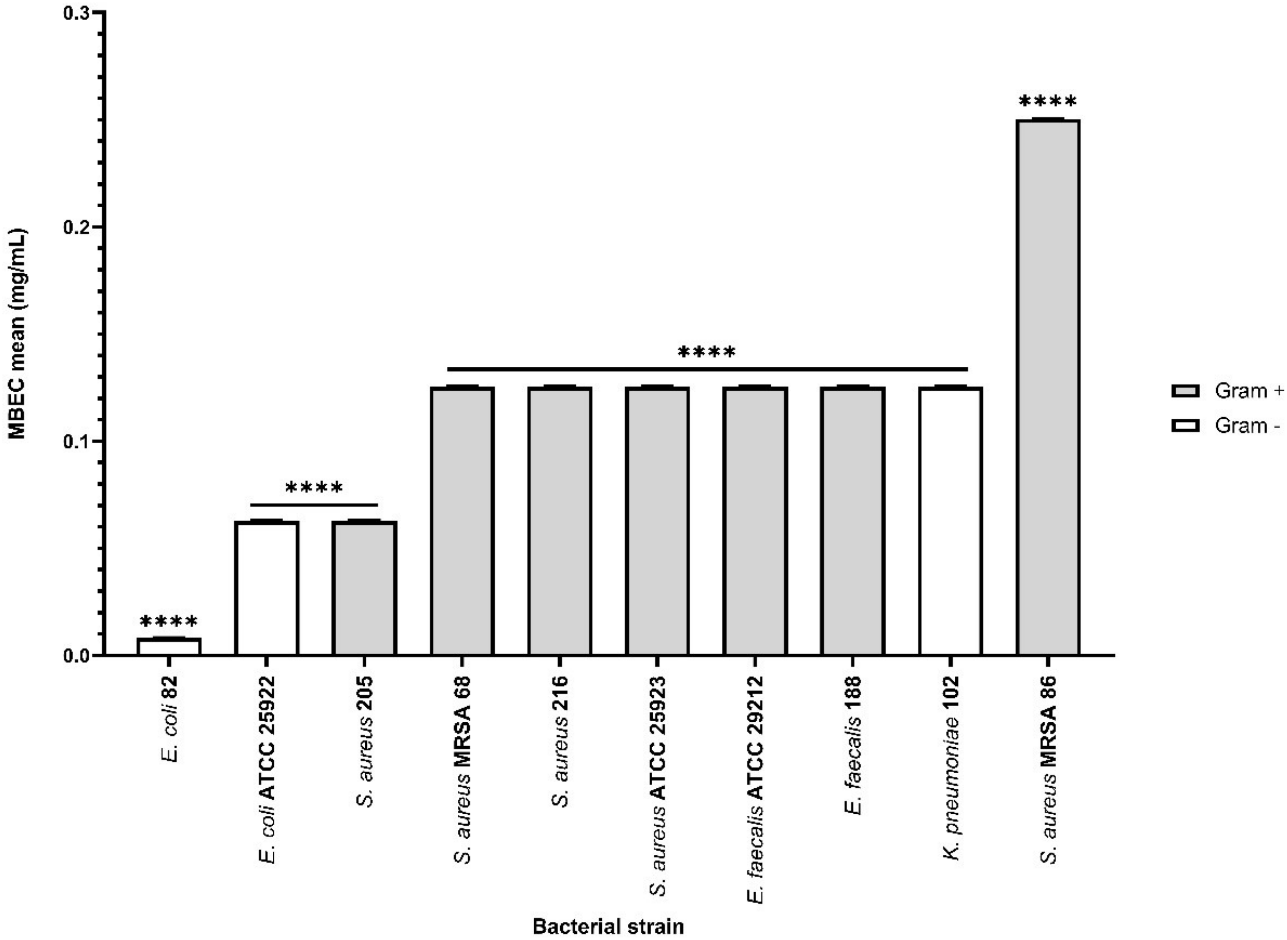
Graphic representation of biofilm modulation of antimicrobial activity of α-T: N combination (1:1) after 24 h incubation; *p < 0.05; **p < 0.01, ***p < 0.001, ****p < 0.0001). Barplots were created with modified data.

Additionally, the inhibitory effect of this combination was also observed in Gram-negative bacterial strains (mean MBEC values between 7.8125 μg/mL and 0.125 mg/mL), with a significant effect for the clinical *E. coli* strain (mean MBEC value of 7.8125 μg/mL) (**Figure 10**). LAB strains didn’t exhibit adhesion capacity to inert substrates.

The results showed that both GA, N and αT individually and in combination did not exhibit antimicrobial activity against LAB strains. This suggests that these compounds do not interfere with the growth of beneficial microorganisms, highlighting their beneficial effects in inhibiting pathogen growth without affecting normal microbiota.

Therefore, these findings support using of natural bioactive compounds as effective agents in HS management, with diverse therapeutic applications. Such compounds could prove to be effective and well-tolerated agents in the treating of chronic wound infections.

### 3.2 Evaluation of the cytokines immunomodulatory pattern

#### 3.2.1 Immune response modulation by interaction of FCs with pathogenic bacteria

The results showed that, following the experimental infection of differentiated THP-1 monocyte-derived macrophages with Gram-negative bacterial strains, significant alterations in cell morphology were observed. Vacuolated cells with rounded or spherical shapes, infiltrated with microbial cells, were detected in both the control group (experimentally infected but untreated cells) and in the presence of FCs. Regarding the experimental infection of differentiated macrophages with Gram-positive bacterial strains, cell viability was strongly affected. This trend was also observed in microscopic evaluations at both 24 and 48 h of incubation, showing dynamic cellular changes. Additionally, cells in various stages of apoptosis, detached from the inert substrate, were identified. On the other hand, co-cultivation of THP-1 cells with LAB strains did not lead to any morphological changes.

**Figure 11:**
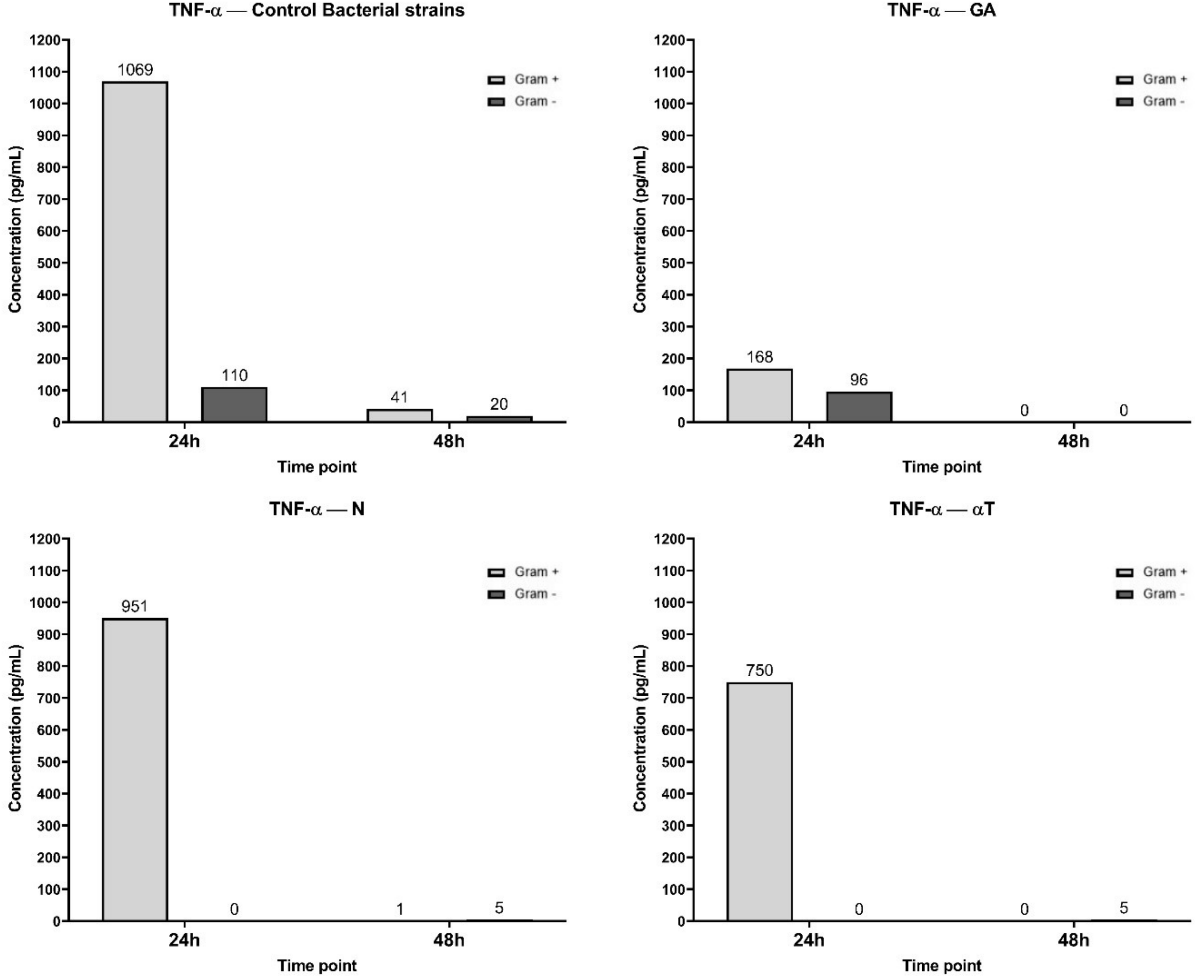
Graphical representation of TNF-α concentration levels at 24h and 48h in response to different bacterial strains in the presence of FCs tested [gallic acid (GA), nerolidol (N) and α-terpineol (αT)].

After 24 h of macrophage cell line infection with pathogenic bacteria suspensions, the signaling cascades leading to TNF-α secretion were activated, as reflected by the high TNF-α levels (**Figure 11**). Phytochemical treatment of the cell line interfered with these pathways, resulting in a reduction in TNF-α production, which was more pronounced when the treatment was performed with subinhibitory concentrations (10 μg/mL) of AG, followed by αT, in case of Gram-positive strains infection. Also, TNF-α levels were undetectable after 24 h when the macrophage cell line infected with Gram-negative bacteria was treated with subinhibitory concentrations of N and αT (**Figure 11**).

**Figure 12:**
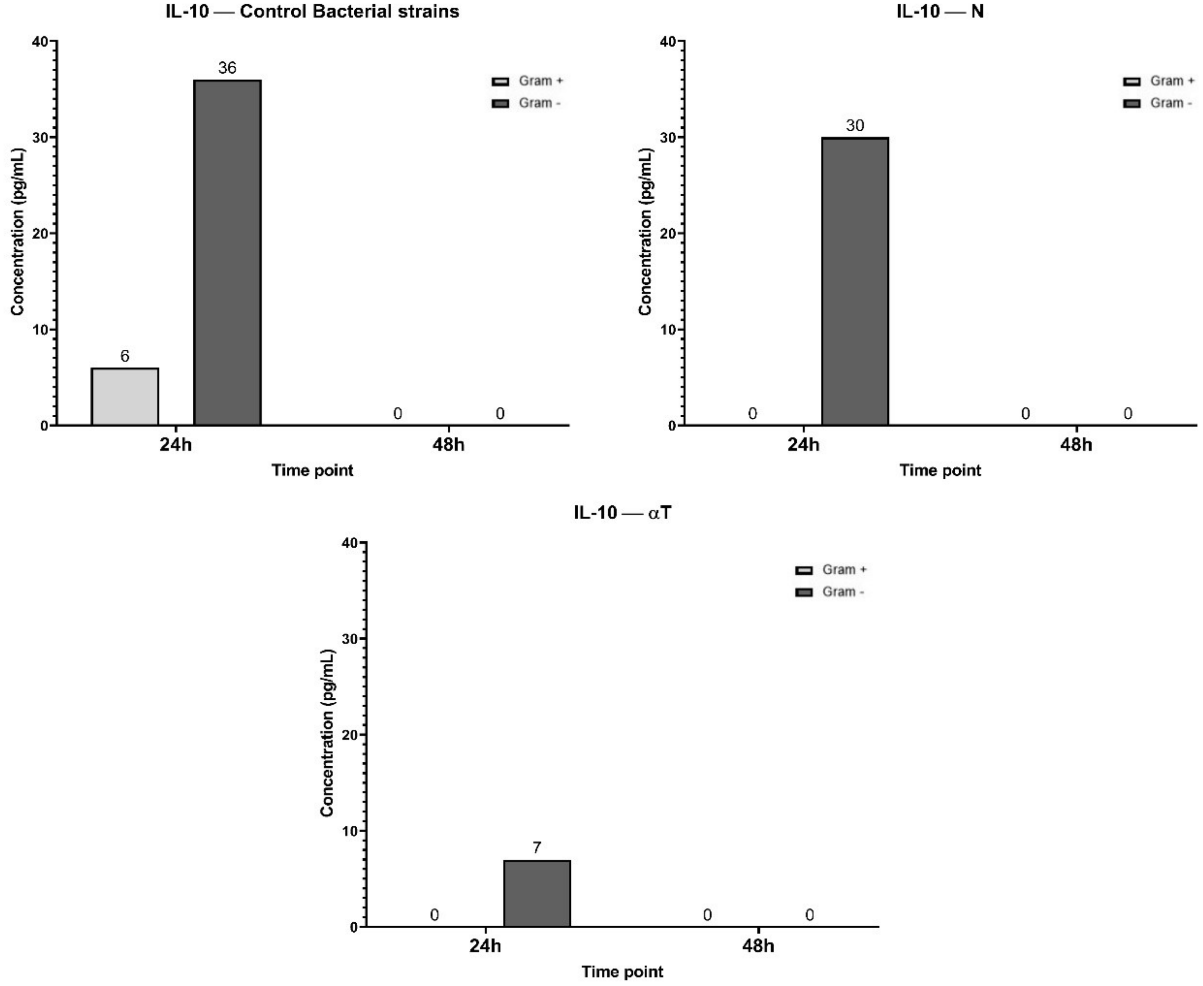
Graphical representation of IL-10 concentration levels at 24h and 48h in response to different bacterial strains in the presence of FCs tested [nerolidol (N) and α-terpineol (αT)]. For GA (gallic acid), ELISA determination showed an undetectable level of IL-10.

TNF-α inhibition could not be correlated with un increasing level of IL-10 after the same experimental treatments (**Figure 12**). IL-10 secretion is significantly higher in Gram-negative (36 pg/mL) infections compared to Gram-positive (6 pg/mL) across all treatments. But in different treatment conditions the IL-10 production is transient and primarily occurs at the 24 h. For GA, ELISA determinations after 24h of incubation showed an undetectable level of IL-10.

**Figure 13:**
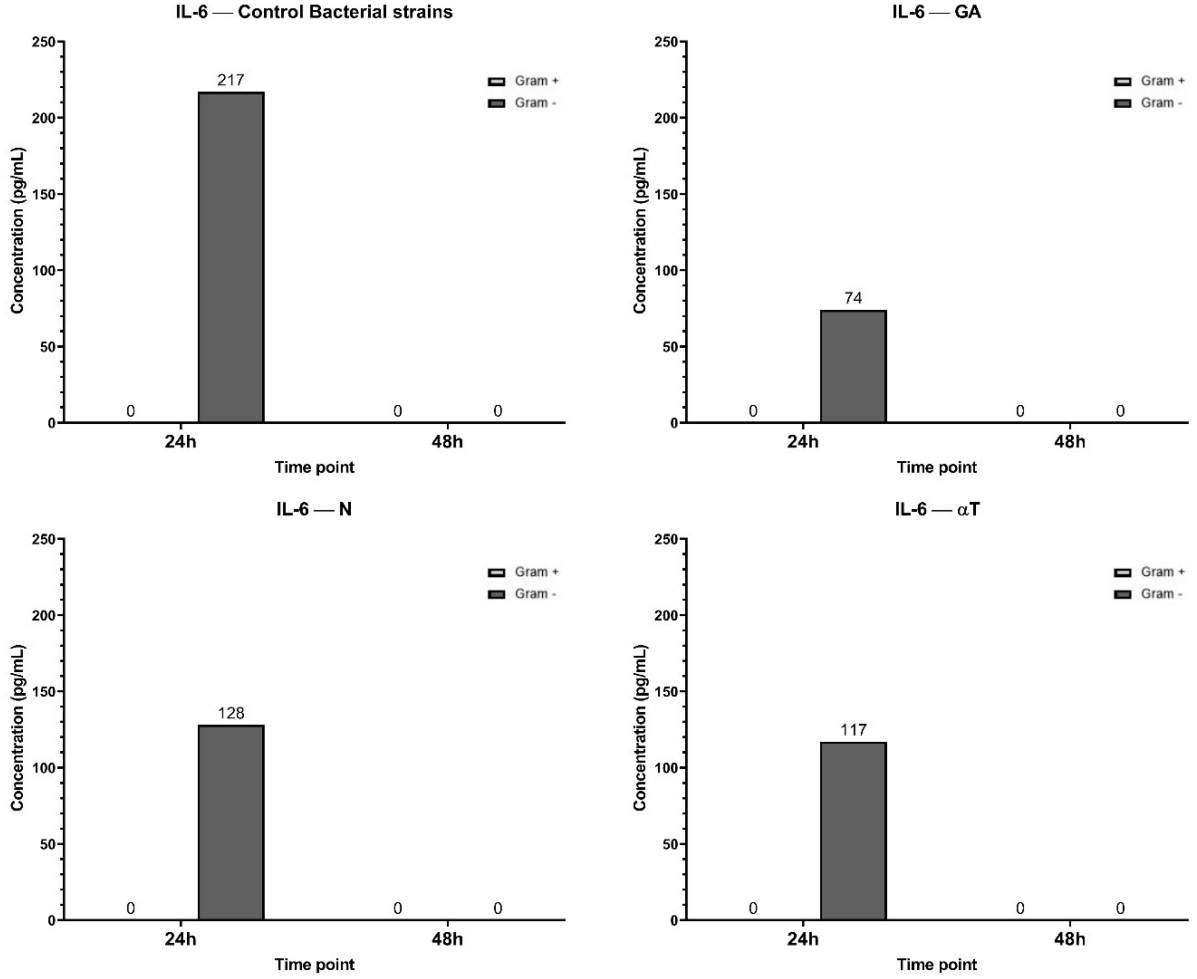
Graphical representation of IL-6 concentration levels at 24h and 48h in response to different bacterial strains in the presence of FCs tested (GA, N and αT).

The highest IL-6 concentration (217 pg/mL at 24h) was observed in the control condition, suggesting a strong inflammatory response upon Gram-negative bacterial infection, but not for Gram-positive (**Figure 13**). By 48 h, IL-6 levels dropped to 0 pg/mL, indicating a transient cytokine response. When the treatment of the infected cell line was performed with GA, the IL-6 levels at 24 h (74 pg/mL) were significantly lower than in the control, suggesting that GA treatment reduced IL-6 production. In αT treatment condition, the IL-6 levels at 24h (117 pg/mL) were lower than the control but higher than GA, suggesting a partial reduction of IL-6 expression. Also, IL-6 expression in N treatment condition was lower than the control (128 pg/mL), but higher than GA and αT, indicating an intermediate effect. By 48 h, IL-6 was completely absent in all conditions, suggesting cytokine clearance or immune regulation over time.

The results suggest that all tested conditions (GA, αT, N) mitigate IL-6 expression compared to the control, with GA showing the strongest suppression. This indicates that these treatments may have anti-inflammatory effects by reducing IL-6 secretion in response to Gram-negative bacterial infection.

**Figure 14:**
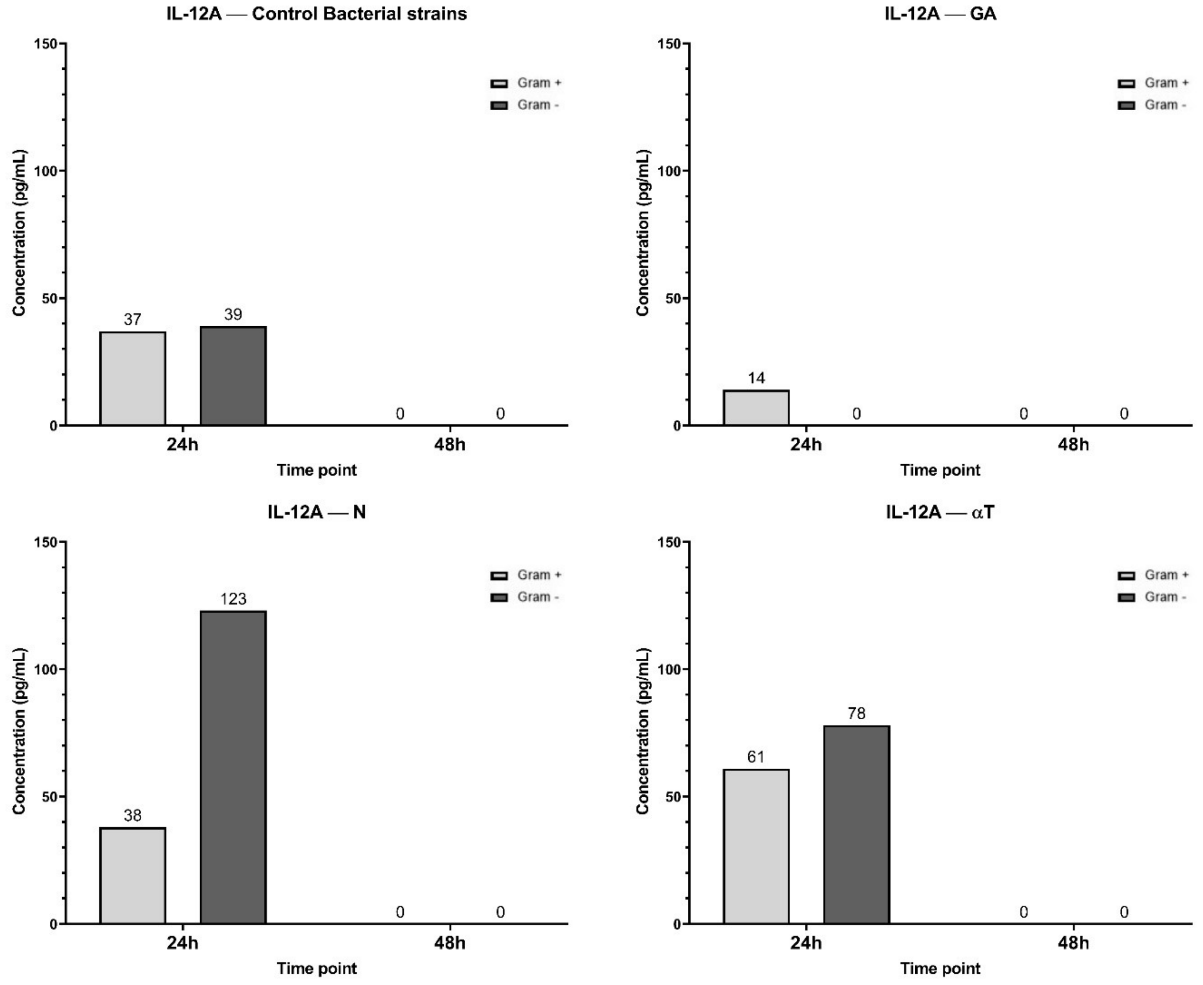
Graphical representation of IL-12A concentration levels at 24h and 48h in response to different bacterial strains in the presence of FCs tested (GA, N and αT).

In the control condition, both Gram-negative and Gram-positive bacterial infections induced a moderate level of IL-12A expression, with undetectable concentrations after 48 h (**Figure 14**). When the infected cell line was treated with N and αT, IL-12A expression was significantly higher than in the control, especially in response to Gram-negative bacterial infections (78 pg/ml and 123 pg/ml, respectively). Treatment of Gram-positive infections led to an increased IL-12A level only with αT (61 pg/ml) compared to the control (37 pg/ml). Under the GA treatment condition, IL-12A levels at 24 h were 0 pg/ml for Gram-negative infections and significantly lower (14 pg/ml) than in the control for Gram-positive infections (37 pg/ml) (**Figure 14**). By 48 h, IL-12A was completely absent in all conditions.

#### 3.2.2 Immune response modulation by interaction of FCs with LAB strains

The expression of ILs was also evaluated using a combination of phytocompounds (at subinhibitory concentrations of 10 μg/mL) with LAB probiotic strains. The concentration of 0.01 mg/mL was selected to avoid cytotoxicity while still enabling detection of immune modulatory effects. Published studies report that GA, N, and αT exhibit cytotoxicity in human keratinocytes and fibroblasts at concentrations typically exceeding 0.05–0.1 mg/mL. Therefore, our chosen dose is within a safe, sub-cytotoxic range suitable for assessing immune-related responses without compromising cell viability.

**Figure 15:**
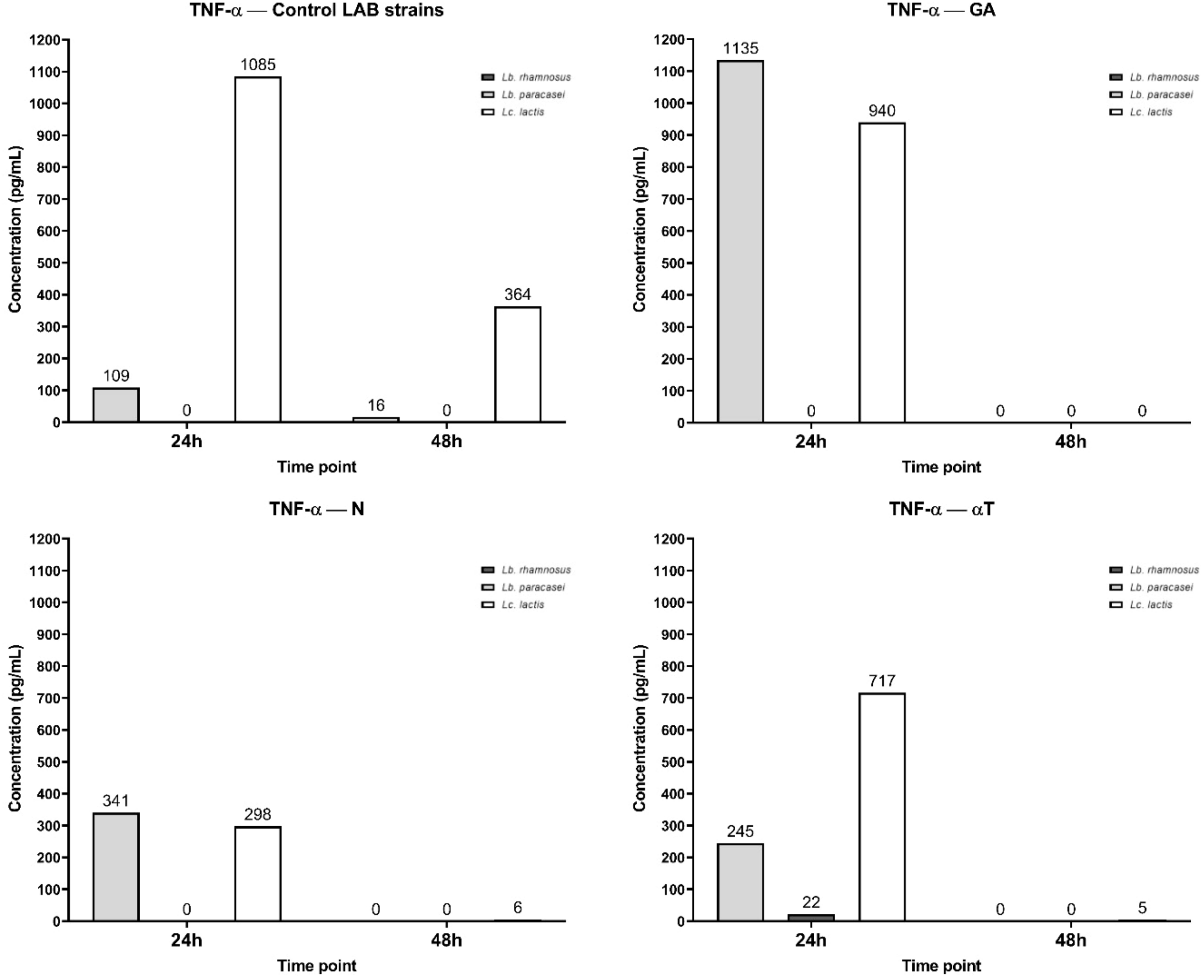
Graphical representation of TNF-α concentration levels at 24h and 48h in response to different LAB strains in the presence of FCs tested (GA, N and αT).

The IL levels were determined after FCs treatment, using a macrophage cell line infected with three different LAB strains as the control. For LAB strains, TNF-α synthesis was inhibited in a strain-dependent manner and influenced by their source of isolation. Specifically, *Lc. lactis*, isolated from dental plaque, induced the highest level of TNF-α synthesis (1085 pg/mL) at 24h, with a prolonged response at 48h (364 pg/mL). *Lb. plantarum*, isolated from sheep cheese, exhibited low TNF-α levels (109 pg/mL at 24h and 16 pg/mL at 48h), indicating that the cytokine response was fading. *Lb. rhamnosus*, isolated from the normal human microbiota (*newborn feces*), did not stimulate TNF-α synthesis (**Figure 15**).

Phytochemical treatment of the cell line interfered with these pathways, *Lc. lactis* infection inducing extremely high TNF-α levels (1135 pg/mL), even higher than the control condition, indicating that GA does not reduce TNF-α secretion but might even enhance the pro-inflammatory response. *Lb. paracasei* also shows an increased TNF-α level (940 pg/mL) compared to the control, suggesting GA enhances inflammation in this condition. *Lc. lactis* remains at 0 pg/mL, consistent with the control. At 48h, a complete absence of TNF-α in all bacteria can be observed, showing a rapid decline, which may indicate that GA enhances early cytokine release but then accelerates immune suppression.

*Lc. lactis* and *Lb. paracasei* infections show moderate TNF-α levels (341 pg/mL and 258 pg/mL, respectively) after N treatment by 24h, significantly lower than the control condition (1085 pg/mL and 109 pg/mL). This suggests that N treatment effectively reduces the inflammatory response. For *Lb. rhamnosus*, the level of TNF-α remains 0 pg/mL, like the control. At 48h, TNF-α levels drop to negligible amounts (0–6 pg/mL), showing that N treatment suppresses long-term inflammation.

After αT treatment, *Lc. lactis* infection shows moderate TNF-α levels (717 pg/mL), significantly lower than the control (1085 pg/mL), indicating, a reduction in inflammation. *Lb. paracasei* infection shows very low TNF-α levels (22 pg/mL), also suggesting strong immunomodulation at 24 h. *Lb. rhamnosus also* remains at 0 pg/mL, like the control. At 48 h, TNF-α levels decrease significantly (0–5 pg/mL), reinforcing αT’s anti-inflammatory effects over time.

**Figure 16:**
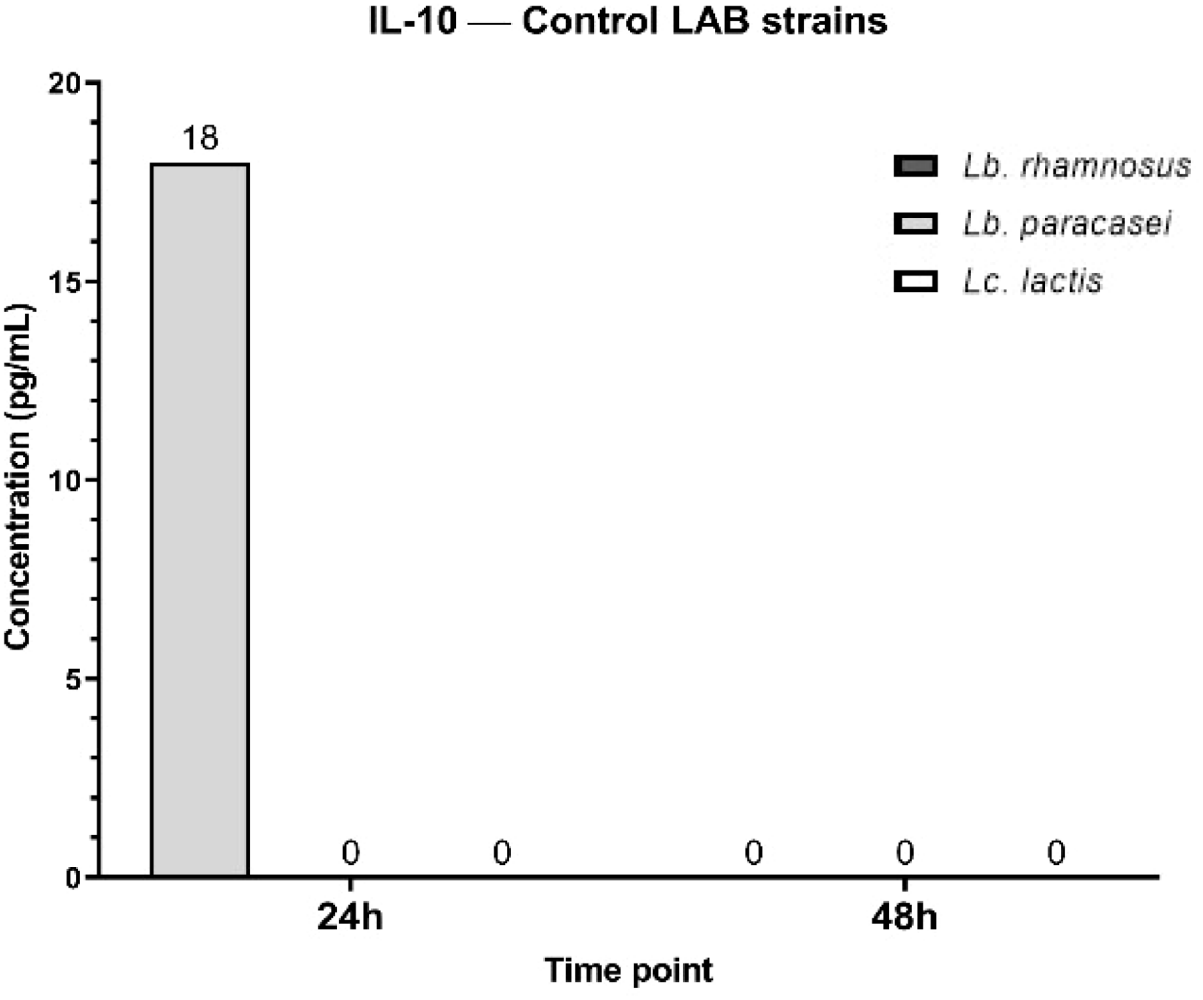
Graphical representation of IL-10 concentration levels at 24h and 48h in response to different LAB strains in the presence of FCs tested (GA, N and αT).

The IL-10 secretory pattern shows that, at 24h, only *Lc. lactis* induces IL-10 production (18 pg/mL), *Lb. paracasei* and *Lb. rhamnosus* show no detectable IL-10 secretion (0 pg/mL). At 48h, IL-10 levels drop to 0 pg/mL for all three bacterial strains, indicating a transient IL-10 response that is present at 2 4h but disappears by 48 h. IL-10 is an anti-inflammatory cytokine that helps regulate excessive immune responses. The transient production of IL-10 at 24 h in response to *Lc. lactis* suggests that this strain temporarily activates an anti-inflammatory response, potentially to counterbalance pro-inflammatory cytokines like TNF-α. The level of IL-10 was undetectable (0 pg/ml) after treatment with tested phytoconstituents (**Figure 16**).

**Figure 17:**
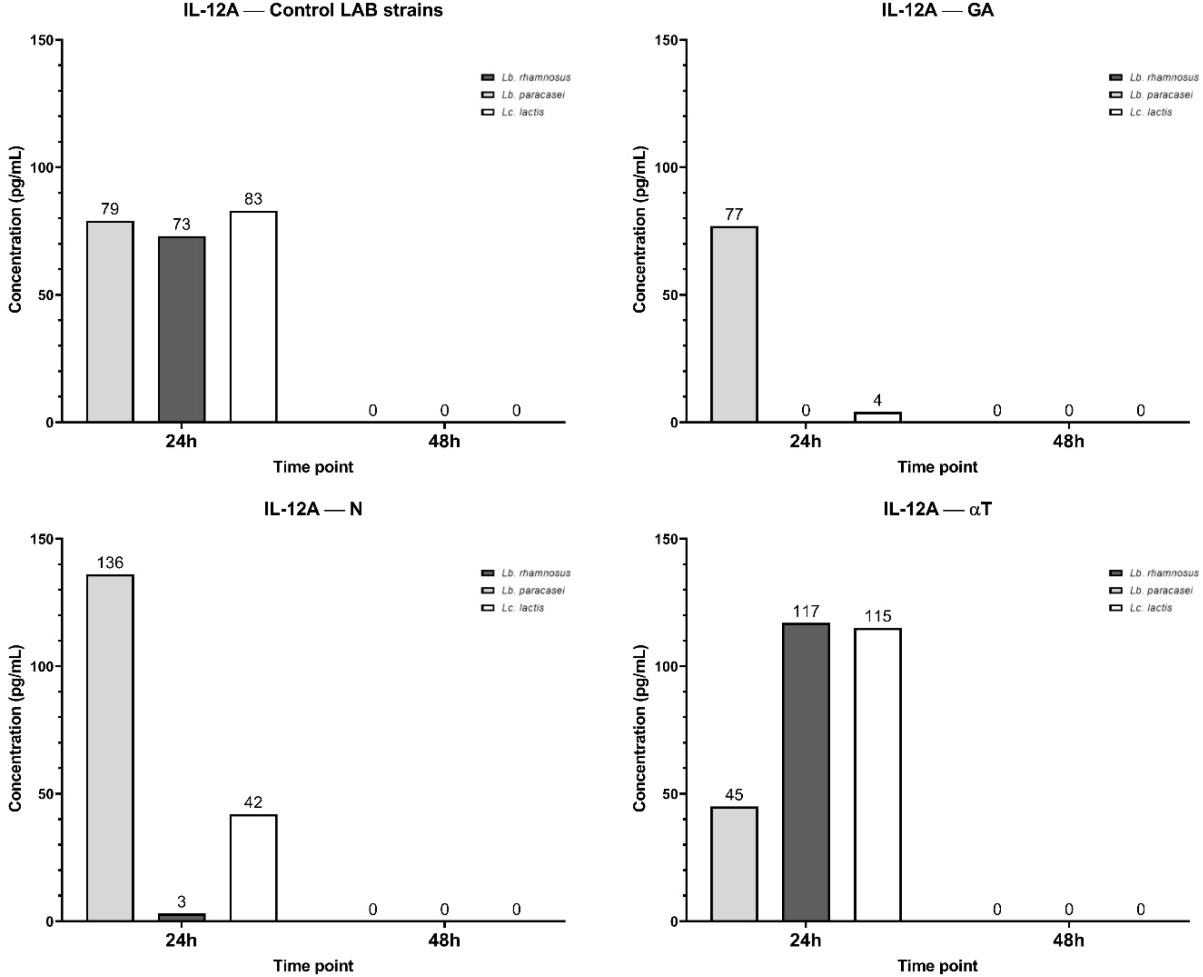
Graphical representation of IL-12A concentration levels at 24h and 48h in response to different LAB strains in the presence of FCs tested (GA, N and αT).

The IL-12A secretory pattern shows that at 24 h, all three LAB strains induce its synthesis at similar concentration levels (**Figure 17**). By 48h, IL-12A secretion is undetectable (0 pg/mL). When *Lb. rhamnosus* and *Lc. lactis* infections were treated with GA at a subinhibitory concentration, IL-12A levels decreased dramatically, whereas no significant reduction was observed for *Lb. paracasei* infection. N treatment exhibited a similar effect on the infected macrophage cell line, with a higher IL-12A level (136 pg/mL) for *Lb. paracasei* compared to the control (79 pg/mL).

In contrast, treatment with αT induced higher IL-12A levels in *Lb. rhamnosus* (117 pg/mL) and *Lc. lactis* (115 pg/mL) infections than in the control, but a lower IL-12A level for *Lb. paracasei* infection (45 pg/mL) (**Figure 17**).

## 4 Discussion

In the context of the antimicrobial resistance (AMR), the development of therapeutic strategies such as those derived from natural bioactive compounds has become a priority for researchers, due to their antimicrobial, anti-adherence and immunomodulatory effects. These compounds target the immune mediators which are responsible for the inhibition of pro-inflammatory cytokine synthesis such as: IL-1β, IL-6, IL-12, IL-17, IL-22, IL-36, TNF-α, IFN- γ, which are commonly involved in the acute and chronic stages of HS. These cytokines are intensely synthetized by macrophages and other immune cells infiltrating the skin lesions (2, 5, 6).

The planktonic (free-floating) growth of microorganisms plays a crucial role in the progression of chronic skin infections. Recent studies suggest that high density of bacterial growth represents a major factor in the evolution of chronic wounds, potentially having a significant impact on infection severity, bacterial virulence and biofilm formation (48).

Biofilms were observed in chronic wound infections, where bacteria embedded in mono- and polymicrobial biofilms exhibit tolerance to common treatments used for local wound care. This not only promotes the persistence of infections but also delays the healing processes (49, 50). In addition, biofilms play an important role in inducing chronic inflammation. Specifically, an increase in inflammatory cytokines such as IL-6, IL-17 A and TNF-α has been observed in wound fluids during infections, especially those associated with biofilm-forming bacteria. The activation of the immune response increases chronic inflammation (51).

Given these challenges, our approach focused on evaluating the antimicrobial properties of three phytochemicals: GA, N and αT against both planktonic and adherent cells. Additionally, our study investigated their immunomodulatory properties in combination with SN probiotic fractions, in order to assess the potential in modulating immune responses. By targeting both microbial growth, biofilm formation and the immune response, these combinations could enhance their bioactivities for the prevention of the progression to chronic stages and treatment of chronic skin infections such as HS.

### 4.1 Evaluation of antimicrobial and antibiofilm activity of FCs

#### 4.1.1 Interaction of FCs with pathogenic bacteria

In line with our results, it was reported that a concentration of 5 mg/mL of GA exhibit a strong inhibiting effect on *P. aeruginosa* monospecific mature biofilms. Additionally, the study showed that GA led to growth inhibition at a MIC of 2.5 mg/mL for both *S. aureus* and *P. aeruginosa* (16). In concordance with this study, Tian et al. demonstrated that GA exhibited antimicrobial activity against nineteen clinically *E. coli* strains and one reference strain, *E. coli* ATCC 25922, with a MIC value of 5 mg/mL (52).

In evaluating the antibacterial activity of GA on *S. aureus* strains IS-58 and K2068, which express the TetK and MepA efflux pumps, it was found that GA displayed an MIC ≥ 1 mg/mL, for both strains. However, it was observed that when is combined with antibiotics, it was generated potentiation of antimicrobial activity. Notably, GA enhanced the effects of tetracycline and ciprofloxacin, suggesting its potential to inhibit efflux pumps activity. These findings highlight the potential of GA as an efflux pump inhibitor, which could improve the efficacy of antibiotics against MDR strains and suggesting that it could be possibly use as an adjuvant in the treatment of difficult-to-treat infections (53).

Other authors also reported similar findings regarding the antimicrobial potential of GA, particularly against MRSA and Methicillin Susceptible *Staphylococcus aureus* (MSSA) strains. The MIC values obtained for GA ranged from 0.2 to 0.4 mg/mL for both MRSA and MSSA. In addition, GA was shown to enhance the effects of β – lactam antibiotics by inducing morphological changes in MRSA strains, generated through cell membrane damage, improving antibiotic’s efficacy against MDR strains and suggesting a potential synergistic effect (54).

Nerolidol has proven their efficiency in the treatment of various bacterial infections including those associated with chronic wounds. Several investigations have been conducted on recognized wound-related strains such as: *S. aureus*, *P. aeruginosa*, *E. coli, E. faecalis* and *K. pneumoniae*, demonstrating its antimicrobial, antibiofilm and antioxidant properties (55–58).

Previous reports demonstrated that N possesses a strong antibacterial behavior against both Gram-positive and Gram-negative bacteria. A study performed recently reported MIC values of 0.5 mg/mL for *P. aeruginosa* and *K. pneumoniae* and 1–4 mg/mL for *S. aureus*, *S. epidermidis*, *E. faecalis* and *E. coli*. Besides, N showed effective antibiofilm properties, inhibiting adherence and biofilm development in a dose-dependent manner and also exhibited antioxidant effects (55).

Santana et al. explored the antimicrobial properties of N both alone and encapsulated in liposomal nano-formulations, on *S. aureus* strains that express efflux pumps: NorA, MsrA and Tet(K). The results revealed that N inhibited the growth of MRSA strains, with MIC values ranging from 32 to 1024 µg/mL. Furthermore, N significantly reduced the MICs of antibiotics, suggesting that N could potentiate the efficacy of them by interfering with bacterial efflux pumps mechanisms. By inhibiting efflux pumps, N increases the intracellular accumulation of antibiotics, increasing their antibacterial properties. All these findings position N as a valuable compound and a promising strategy for use as an adjuvant in the treatment of chronic infections, especially when it is combined with conventional antibiotics (56).

In concordance with the results obtained in our study, αT demonstrated superior efficacy against bacterial strains including: *E. coli, S. aureus*, MRSA, *P. aeruginosa, Bacillus cereus, Streptococcus mutans* and *Salmonella typhimurium* (31, 33, 59).

Researchers in biomedical field also revealed that terpenes such as N and αT enhance the absorption of lipophilic molecules and improving permeability in the cutaneous tissue. Their lipophilicity and ability to interact with skin lipids molecules promote drug delivery. These results make terpenes promising candidates for transdermal delivery systems, potentially useful in wound healing formulations which could be able to enhance the efficacy of therapeutic agents (60).

A study conducted by Johansen and Sergere (31) demonstrated that a combination of two terpenoid isomers αT and terpinen-4-ol inhibit microbial growth and proliferation of ESKAPE pathogens, making this combination a strong natural candidate for antimicrobial applications. Additionally, this combination revealed that the doses of these two isomers could potentially be reduced when they are used together, reducing potential side effects such as: toxicity, allergenicity and resistance mechanisms, while maintaining antibacterial behavior. These findings align with our study, where the combination of αT and N, two terpenoids, exhibit promising antimicrobial, anti-adherence and immunomodulatory properties. The cumulative effects of these two compounds could potentially provide an effective therapeutic solution for HS therapy associated with pathogenicity and inflammation, reducing the risks associated with higher concentrations when they are used as individual compounds.

Still, according to our knowledge, there are no references available to compare our results regarding the antimicrobial and antibiofilm activity of αT, N & GA combinations with potential therapeutic applications in the treatment of HS. Additionally, no results have been reported yet on the probiotic – natural bioactive compound combinations effects on the modulation of inflammatory responses.

#### 4.1.2 Interaction of FCs with LAB

Interestingly, a study performed on phenolic compounds such as: catechin, gallic acid (GA), vanillic acid, ferulic acid and protocatechuic acid present in fruits and vegetables provides selective growth effects on both beneficial and pathogenic microorganisms. These compounds promoted the growth of beneficial lactobacilli such as *Lb. rhamnosus* GG and *Lactobacillus acidophilus*, while inhibiting the growth of *E. coli* and *Salmonella typhimurium.* The selective antimicrobial activity holds significant potential for the development of nutraceutical products (61). Moreover, combining natural bioacitive compounds with probiotics, which are known as pathogen antagonists due to their ability to produce antimicrobial molecules such as bacteriocins (62), could amplify their bioactivities. The interaction between LAB probiotic strains and natural bioactive compounds represents a promising strategy in the management of chronic wounds such as HS, not only by inhibiting growth of pathogens, but also promotes the proliferation of beneficial microorganisms that modulate inflammation, offering a promising approach, without affecting normal microbiota (61).

### 4.2 Evaluation of the cytokines immunomodulatory pattern

#### 4.2.1 Immune response modulation by interaction of FCs with pathogenic bacteria

The specific characteristics of the bacteria appear to alter the immune response to Gram-positive bacterial components. The distinct cytokine profiles triggered by Gram-positive and Gram-negative bacteria may help optimize the clearance of bacteria with different cell wall structures (63). The data presented in this study suggests intricate immune modulation of macrophage responses to bacterial infections, particularly in relation to TNF-α, IL-6, IL-10, and IL-12A cytokine profiles. The experiment investigated the effects of different treatments (GA, αT, N) on macrophage responses to Gram-positive and Gram-negative bacterial infections and their interactions with these cytokine pathways.

The pathogenic bacteria infections—both Gram-positive and Gram-negative—activated a strong inflammatory response, as reflected by the elevated TNF-α levels after 24 h. This cytokine was significantly higher in infections involving Gram-negative bacteria compared to Gram-positive bacteria. The treatments with GA, αT and N led to varied effects on TNF-α production. For Gram-negative infections, treatment with subinhibitory concentrations of N and αT led to undetectable TNF-α levels, suggesting a complete suppression of macrophage inflammatory responses in these conditions. The differential effects between Gram-positive and Gram-negative infections suggest that FCs interact with distinct bacterial components (lipoteichoic acid vs. LPS), especially since N and αT, alone or in combination, inhibited the ability of the pathogenic strains to adhere and develop mature biofilms (as suggested in **Figures 5** and **6**). By 48 h, TNF-α levels were undetectable across all treatments, suggesting a time-dependent cytokine clearance or immune regulation.

Pathogenic Gram-negative bacterial infections induced a strong IL-6 response at 24 h, indicating a robust pro-inflammatory reaction. In contrast, IL-6 production following Gram-positive bacterial infections was much lower. The treatments significantly reduced IL-6 levels at 24 h, with gallic acid showing the strongest suppression. This suggests that the tested compounds have anti-inflammatory effects that limit IL-6 secretion, particularly in response to Gram-negative bacterial infections. By 48 h, IL-6 levels dropped to zero in all treatment conditions, supporting the idea that the inflammatory response was transient and under tight immune regulation. The role of GA in reducing inflammation by regulating the expression level of genes involved in neutrophil functionality has been demonstrated. Thus, in milk phagocytes, GA induced a reduction in the expression of proinflammatory genes (*IL1B, IL6, TNF*) (64) by downregulating nuclear factor-κB (65).

Anti-inflammatory cytokine involved in wound healing and suppressing immune system activators is IL-10 (66). In our experiment, the IL-10 levels were higher in response to Gram-negative bacterial infections compared to Gram-positive ones. However, the secretion of IL-10 was transient, peaking at 24 h and returning to baseline levels by 48 h. IL-10 serves as an anti-inflammatory cytokine that likely functions to counterbalance the pro-inflammatory effects of TNF-α (67). Inhibition of IL-10 secretion was observed under all treatment conditions, with levels undetectable in all tested conditions. This suggests that the phytochemical treatments may suppress the anti-inflammatory responses typically mediated by IL-10, although IL-10 is naturally transient in response to these infections.

IL-12A levels were moderately induced across both Gram-negative and Gram-positive bacterial infections, though it remained at low levels and became undetectable by 48 h. GA treatment reduced IL-12A levels in both Gram-negative and Gram-positive infections, with the most notable suppression seen in Gram-negative infections, where IL-12A levels dropped to zero. This suggests that GA might not only suppress pro-inflammatory cytokines like TNF-α but could also dampen IL-12A, which is typically involved in promoting Th1 immune responses (68).

#### 4.2.2 Immune response modulation by interaction of FCs with LAB

The study evaluated the immune response modulation by different LAB probiotic strains in combination with FCs (GA, N, and αT) by measuring TNF-α, IL-10, and IL-12A levels in a macrophage cell line. The findings provide insight into how probiotics and phytoconstituents influence pro-inflammatory and anti-inflammatory pathways, which has implications for their potential therapeutic role in Hidradenitis Suppurativa (HS) treatment.

LAB strain-dependent TNF-α synthesis was observed, with the highest levels seen in *Lc. lactis* (from dental plaque), followed by *Lb. plantarum* (from sheep cheese), while *Lb. rhamnosus* (from newborn feces) did not induce TNF-α secretion. The source of isolation appears to influence the strain’s immunogenicity, as *Lc. lactis* triggered strong inflammatory responses compared to other strains.

Phytochemical treatment with GA significantly enhanced TNF-α levels in *Lc. lactis* and *Lb. paracasei* infections, suggesting that this compound may exacerbate inflammation rather than reducing it in some contexts. However, by 48 h, TNF-α levels dropped to negligible amounts for all strains, indicating that FC may trigger an early inflammatory response but then accelerate immune suppression over time.

N treatment significantly reduced TNF-α levels, especially in *Lc. lactis* and *Lb. paracasei* infections, suggesting a strong anti-inflammatory effect. αT also reduced TNF-α expression, with *Lb. paracasei* showing the most significant reduction, indicating potential immunomodulatory properties that may be beneficial for HS treatment.

Regarding the anti-inflammatory response, only *Lc. lactis* induced IL-10 production at 24h, while *Lb. paracasei* and *Lb. rhamnosus* did not. After 48h, IL-10 levels dropped to 0 pg/mL in all conditions, suggesting a transient anti-inflammatory response at 24h that is not sustained. Future studies could explore combining LAB strains with other anti-inflammatory phytochemicals that enhance IL-10 secretion, such as resveratrol or quercetin (69, 70). Some studies on mast cells (MC), as innate immune skin-resident cells, strongly support the role of resveratrol (a phenolic compound also) on local MC stabilization, accompanied by decreased levels of chemokines and its infiltration in otherwise inflamed skin (70).

IL-12A is involved in Th1 immune responses, which contribute to chronic inflammation in HS (68). The transient nature of IL-12A response suggests that LAB strains activate immune pathways early but do not sustain inflammation over time.

These findings suggest that the interplay between probiotics and phytochemical treatments is strain-dependent, with certain strains being more responsive to immunomodulation than others. The variation in cytokine responses to different LAB strains further underscores the importance of strain-specific interactions in modulating the immune system. This could have implications for designing targeted therapies using probiotics or FCs for inflammatory conditions. A possible application for HS treatment may consider the combination of *Lb. paracasei* with N or αT that could provide balanced immune modulation, reducing excess inflammation while maintaining protective immune responses. Future therapies must focus on personalized probiotic treatments based on individual microbiome profiles and immune response patterns.

## 5 Limitations

This study was primarily conducted on *in vitro* models and future studies must be conducted *in vivo* to evaluate the therapeutic efficacy and pharmacokinetics of these compounds in relevant animal models. Additionally, while promising antimicrobial and immunomodulatory effects were observed, further research is indeed to develop and optimize topical formulations, including assessments of compound stability, skin penetration and cytotoxicity.

Although the selected pathogens are representative, they do not fully capture the diversity of microbial species associated with HS and other skin infections; therefore, broader microbiological profiling is required to gain a more comprehensive understanding of the complex microbial communities involved. Moreover, deeper mechanistic studies should be undertaken to reveal the molecular pathways involved in the observed bioactivity, optimize dosing applied and investigate the potential interactions between phytocompounds and probiotic products.

Although the combinations of gallic acid, α-terpineol, and nerolidol were selected based on their complementary mechanisms of action and promising *in vitro* effects, one of the main limitations of this study is the absence of synergy quantification using standard methods such as fractional inhibitory concentration (FIC) or FIC index (FICI) assays. Future studies should incorporate quantitative approaches to validate potential synergistic interactions, as well as expanded dose–response analysis and time-kill kinetics, to better characterize the nature and reproducibility of the observed effects.

Data analysis limitations were identified in the experiments corresponding to the evaluation of antimicrobial and antibiofilm activity of FCs or to the evaluation of the cytokines immunomodulatory pattern. The former research interest generated perfect data with no variation between the replicates, requiring the introduction of artificial variance (< 6.5%) in order to allow statistical testing in the form of unpaired Student’s t tests, as well as ANOVA with *post-hoc* Tukey’s multiple comparisons test. The latter research interest generated multiple low absorbance readings, which resulted in negative cytokine concentrations calculations. Negative data was excluded from further analysis. Mean cytokine concentrations were used for Gram-positive or Gram-negative strains, which did not allow statistical testing groups of interest (n = 1).

## 6 Conclusions

The compounds tested in this study showed promising preclinical antimicrobial properties, sometimes complementary action, and the potential to modulate inflammatory pathways *in vitro.*

The results of the study highlighted the inhibitory effect of the natural bioactive compounds (gallic acid, nerolidol and α-terpineol) on the growth, proliferation and biofilm development of HS-related pathogenic agents. The combination of α-terpineol and nerolidol demonstrated antimicrobial activities specifically against Gram-positive species such as: *S. aureus* and *E. faecalis*, both standard and clinical strains, and anti-biofilm effects with a remarkable impact against all strains tested.

These results suggest that such phytoconstituents may be considered valuable candidates for further investigation in skin infections and inflammation-related disorders, including HS. However, the current evidence remains preliminary, and for proof of therapeutic efficacy, future studies are needed for developing and testing suitable topical formulations, assessing factors like compounds’ stability and skin penetration, testing in animal experimental models, and establishing potential side effects.

Combining probiotics with phytoconstituents provides a promising strategy for cytokine modulation, with potential applications in managing infections and inflammatory diseases. The effects of these two components can help fine-tune immune responses, suppress excessive inflammation, and promote a balanced immune profile. This combination may be particularly beneficial in conditions where the immune system is dysregulated.

Therefore, the interactions observed between probiotics and phytocompounds in the present study form the basis for further exploration in order to understand their action mechanisms and optimize the tested combinations. Remarkably, the association of *Lactobacillus paracasei* with nerolidol or α-terpineol should be further evaluated in animal experimental models, focusing on IL-10 induction, biofilm inhibition, and immune homeostasis.

Ongoing research into the antimicrobial and immunomodulatory properties of natural bioactive compounds is opening new opportunities in the biomedical field. These compounds demonstrate valuable potential for developing effective and well-tolerated topical treatments, positioning them as promising candidates for future therapeutic strategies focused on the management of HS and related skin conditions.

## Data Availability

The original contributions presented in the study are included in the article/**Supplementary Material**. Further inquiries can be directed to the corresponding author.
